# Wogonin, a bioactive flavonoid from *Scutellaria baicalensis*, alleviates alcoholic pancreatitis via AMPK-mediated TFEB activation

**DOI:** 10.1016/j.jare.2025.11.006

**Published:** 2025-11-10

**Authors:** Zhen Qin, Xiaohong Sun, Jingmin Zeng, Guorong Li, Haixia Wang, Shujun Chen, Shiyun Tan, Lin An, Caiyan Wang, Liming Wang, Jinping Wang, Xiaowen Ma, Zhongxiang Zhao, Xiaojuan Chao, Zhongqiu Liu, Shaogui Wang

**Affiliations:** a State Key Laboratory of Traditional Chinese Medicine Syndrome, Chinese Medicine Guangdong Laboratory (Hengqin Laboratory), International Institute for Translational Chinese Medicine, School of Pharmaceutical Sciences, Guangzhou University of Chinese Medicine, Guangzhou, Guangdong, China; b Department of Pharmacy, Shenzhen Children’s Hospital, Shenzhen, China; c Key Laboratory of Chinese Medicinal Resource from Lingnan (Guangzhou University of Chinese Medicine), Ministry of Education, Guangzhou, China; d School of Biomedical Science, Hunan University, Changsha, Hunan, China; e Department of Pharmacy, Shenzhen Second People’s Hospital, Shenzhen, China; f Translational and Clinical Research Institute, Faculty of Medical Sciences, Newcastle University, Newcastle Upon Tyne, UK; g Institute of Precision Medicine, The First Affiliated Hospital, Sun Yat-sen University, Guangzhou, Guangdong, China

**Keywords:** TFEB, Autophagy, Alcoholic pancreatitis, Zymogen granule

## Abstract

**Introduction:**

Transcription factor EB (TFEB) deficiency contributes to insufficient autophagic degradation, resulting in zymogen granule (ZG) accumulation and subsequent premature activation, which underlies the pathogenesis of alcoholic pancreatitis (AP). Despite this mechanistic insight, pharmacological activation of TFEB via plant-derived agents remains an underdeveloped therapeutic avenue.

**Objectives:**

This study aims to investigate Wogonin’s role as a key *Scutellaria baicalensis* (Huangqin, HQ) component in protecting against AP, with specific focus on TFEB-mediated autophagy activation.

**Methods:**

Using TFEB-AcGFP and mRFP-GFP-LC3 reporter systems, we assessed Wogonin-enriched HQ extracts on TFEB nuclear translocation and autophagic flux. Pharmacological and genetic approaches were combined to established HQ/Wogonin’s effects on alcohol-induced pancreatic injury through TFEB-mediated autophagic restoration. Structure-based virtual screening and molecular docking techniques were employed to predict the binding activity of Wogonin with upstream kinases regulating TFEB. Prioritized interactions underwent biophysical validation via surface plasmon resonance (SPR), isothermal titration calorimetry (ITC) and cellular thermal shift assay (CETSA) assays.

**Results:**

HQ/Wogonin enhanced TFEB nuclear translocation and transcriptional activity, thereby restoring autophagic degradation to exert protection against alcohol-induced ZG accumulation and pancreatic injuries. Acinar TFEB knockout abolished HQ’s protective effects against AP. Mechanistic studies revealed Wogonin directly bound with AMPK (AMP-activated protein kinase) to promote its protein stability, leading to improved TFEB activation.

**Conclusion:**

This study establishes Wogonin as the pivotal bioactive driver behind HQ’s therapeutic efficacy, orchestrating AMPK-TFEB-autophagy coordination to mitigate AP. Atomic-level resolution of Wogonin’s adenine-mimetic binding to AMPK provides novel therapeutic strategies for developing kinase-stabilizing botanicals targeting TFEB activation to treat pancreatitis.

## Highlights

• Wogonin serves as a novel TFEB activator rescuing alcohol-induced pancreatitis.
• Wogonin’s protection against alcoholic pancreatitis is TFEB-dependent.
• Wogonin pioneers kinase-stabilizing phytochemical strategy with AMPK interaction.
• Wogonin links herbal medicine to autophagy-targeted therapy for pancreatitis.

## Graphical abstract

**Figure:**
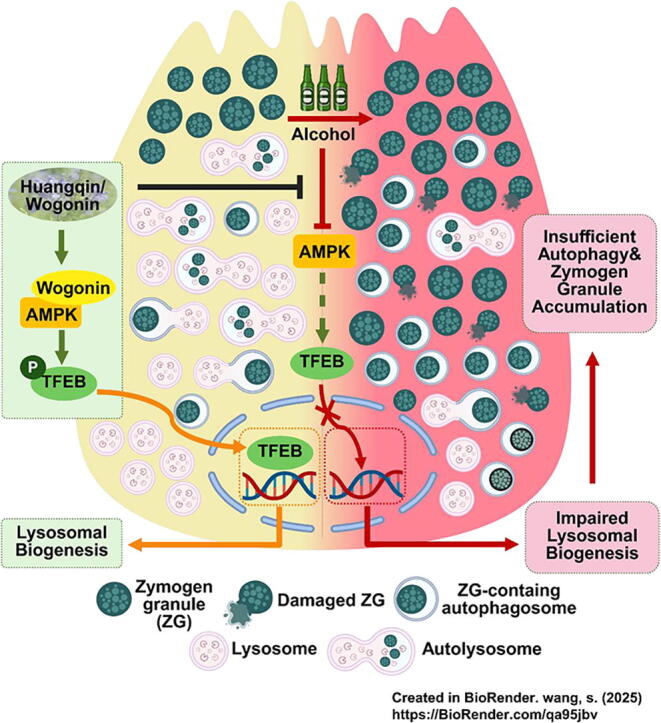

## Introduction

Pancreatitis, an inflammatory disorder of the pancreas, remains a significant clinical challenge due to its complex pathogenesis and limited effective therapies. Chronic alcohol consumption is a well-documented risk factor for both acute and chronic pancreatitis, leading to pathological alterations such as elevated digestive enzyme levels, destabilized zymogen granules (ZGs) in pancreatic acinar cells, and premature enzyme activation. Despite the prevalence of alcohol abuse, only a fraction of individuals develop pancreatitis, implicating intracellular protective mechanisms that counteract alcohol-induced cellular stress.

Studies have established insufficient autophagy as a critical contributor to acute pancreatitis pathogenesis [[1], [2], [3]]. Autophagy, an evolutionarily conserved lysosome-dependent catabolic process, is essential for maintaining acinar cell homeostasis through selectively clearance of damaged endoplasmic reticulum (ER) to preserve ER integrity, mitigate ER stress, and eliminate fragiled ZGs, thereby preventing pathological trypsinogen activation [4,5]. Our previous work demonstrated that chronic-plus-acute alcohol exposure disrupted TFEB, the master regulator of autophagy and lysosomal biogenesis, leading to defective autophagy and AP progression in mice. Specifically, acinar cell-specific TFEB knockout mice developed mild pancreatic edema on standard diets but exhibited severe pancreatitis when fed an ethanol-containing Lieber-DeCarli diet. Conversely, TFEB overexpression ameliorated alcoholic pancreatic injuries [5], making it a potential intervention target for AP. However, the therapeutic potential of pharmacological TFEB activation remains unexplored, and clinically relevant TFEB agonists are currently unavailable.

The activity of TFEB is determined by its subcellular localization, which mainly depends on its phosphorylation status. The mechanistic target of rapamycin complex 1 (mTORC1) is the most well-studied upstream regulator that phosphorylates TFEB at serine 122, 142 and 211 [6]. The adenosine monophosphate-activated protein kinase (AMPK), an energy-sensing hub, was reported to phosphorylate folliculin-interacting protein 1 (FNIP1) to disrupt folliculin-RagC-mTORC1 docking, leading to mTORC1 inhibition and subsequent TFEB nuclear translocation and activation [7]. Moreover, AMPK phosphorylates TFEB/TFE3 directly at serine 466, 467 and 469 to boost their transcriptional activity, especially during stress or chemotherapy [8]. AMPK activity was suppressed during cerulein-induced AP [9,10]. However, whether AMPK inhibition contributed to the disruption of TFEB in AP is unclear.

The dried root of *Scutellaria baicalensis* Georgi (Huang-Qin, HQ), a traditional Chinese medicinal herb, has been demonstrated anti-inflammation properties in preclinical studies [[11], [12], [13]]. Notably, HQ is extensively incorporated into classical Chinsese formulas for pancreatitis management, including Dachaihu, Qingyi and Chaiqinchengqi decoctions. The main pharmacological active components of HQ include Wogonin, baicalin, baicalein, wogonoside, oroxylin A, et al [14]. These components have demonstrated a wide range of pharmacological activities, including anti-inflammatory, antioxidant, anti-cancer, antiviral, antibacterial, neuroprotective, cardioprotective and hepatoprotective effects [15]. Despite its empirical therapeutic use, the molecular mechanisms underlying HQ’s protective effects against pancreatitis are poorly characterized.

In this study, we revealed that Wogonin-enriched HQ promote TFEB nuclear translocation and transcriptional activity, thereby restoring autophagic degradation and conferring protection against alcohol-induced pancreatitis in a TFEB-dependent manner. Mechanistically, Wogonin directly interactes with AMPK to enhance its protein stability, facilitating TFEB activation and autophagy induction.

## Results

### HQ promotes TFEB nuclear translocation and transcriptional activities

**Fig. 1:**
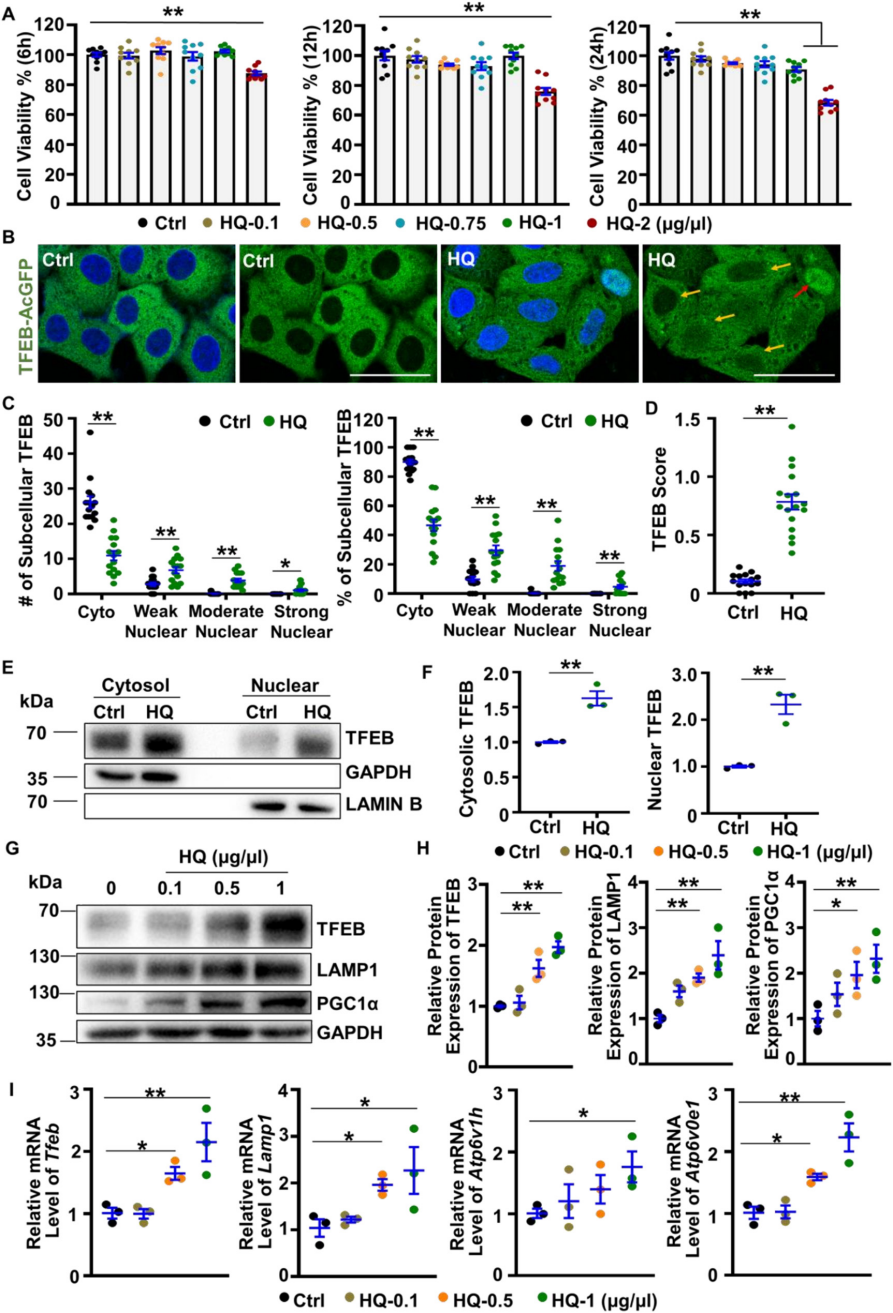
HQ activates TFEB *in vitro*. (A) Cell viability was assessed using CCK-8 assay following treatment with HQ. (B) TFEB distribution was detected in TFEB-acGFP expressing Hela cells. Yellow or red arrow denotes moderate or strong nuclear TFEB, respectively. Scale bar = 50 μm. (C) The number and percentage of cytosolic and nuclear TFEB were quantified, data shown are mean ± SEM (n = 3). More than 400 cells were counted in each group. **p < 0.01, *p < 0.05 by Student *t* test. (D) TFEB score was calculated based on the subcellular distribution of TFEB. (E)Immunoblotting analysis using cytoplasmic and nuclear fractions from HQ-treated Hela cells. (F) Densitometry analysis was performed for cytosolic and nuclear TFEB, data shown are mean ± SEM (n = 3). **p < 0.01, *p < 0.05 by Student *t* test. (G) Total cell lysates were subjected to immunoblotting analysis. (H) Densitometry analysis was performed for TFEB, LAMP1 and PGC1α, data shown are mean ± SEM (n = 3). **p < 0.01, *p < 0.05 by one way ANOVA. (I) mRNA was extracted and used for qPCR. Results were normalized to *Gapdh* and expressed as fold change compared to control. Data are mean ± SEM (n = 3). **p < 0.01, *p < 0.05 by one way ANOVA. (For interpretation of the references to colour in this figure legend, the reader is referred to the web version of this article.)

We systematically investigated the regulatory effects of HQ on TFEB dynamics *in vitro*. Cell Counting Kit-8 (CCK8) assay revealed significant growth inhibition at 2 μg/μl HQ across all timepoints (6/12/24 h), whereas dose ≤ 1 μg/μl maintained normal proliferation rates (Fig. 1A). Using a Hela cell line stably expressing TFEB-AcGFP, we observed substantial nuclear accumulation of TFEB following 6 h treatment with HQ at 1 μg/μl (Fig. 1B–D). Subcellular fractionation analysis confirmed the enhanced nuclear accumulation of TFEB, coupled with upregulation of endogenous TFEB expression in both cytoplasmic and nuclear compartments (Fig. 1E–F). Subsequent immunoblotting and RT-qPCR analyses demonstrated dose-dependent upregulation of TFEB protein and mRNA, paralleled by coordinate induction of lysosomal markers including LAMP1 (Fig. 1G–I). Moreover, HQ induced TFEB nuclear translocation and transcriptional activity in a time-dependent manner (Supplementary Fig. 1). These data collectively indicate HQ effectively activates TFEB signaling and enhances its transcriptional regulatory functions.

### HQ promotes autophagic flux

**Fig. 2:**
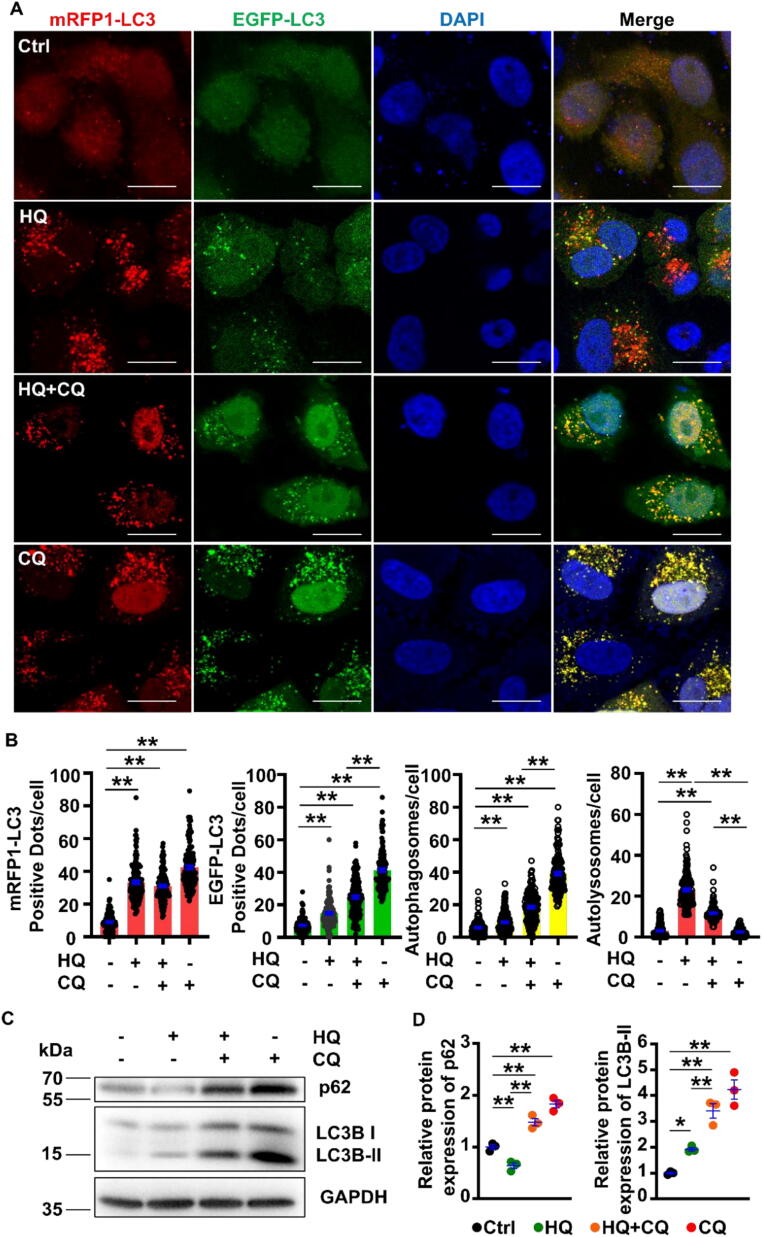
HQ promotes autophagy *in vitro*. (A) HeLa-EGFP-mRFP-LC3 reporter cells treated with HQ (1 μg/μl) ± CQ (25 μM) for 6 h. Representative microscopic images are shown. Scale bar = 20 μm. (B) The number of mRFP-LC3, EGFP-LC3, autophagosome and autolysosome puncta were quantified. Data are mean ± SEM (n = 3). **p < 0.01, *p < 0.05 by one way ANOVA. 150 cells were counted in each group. (C) Total cell lysates were subjected to immunoblot analysis. (D) Densitometry quantification was performed for p62 and LC3II (normalized to loading control GAPDH). Data are mean ± SEM (n = 3). **p < 0.01, *p < 0.05 by one way ANOVA.

TFEB orchestrates a transcriptional network coordinating autophagosome-lysosome fusion efficiency and lysosomal biogenesis [16,17]. We next determined whether HQ-induced TFEB activation governs autophagic flux regulation. Given that autophagy progression relies on sequential maturation from autophagosomes to degradative autolysosomal compartments, we generated Hela cells stably expressing tandem fluorescent EGFP-mRFP-LC3. Confocal microscopy revealed predominantly diffuse EGFP-mRFP-LC3 signals with limited puncta formation under basal conditions (Fig. 2A). Remarkably, HQ treatment induced substantial formation of autophagosomes and autolysosomes, particularly increasing autolysosomes (mRFP-LC3 positive puncta) numbers (Fig. 2, Fig. 2). Moreover, HQ treatment significantly increased the autophagic flux ratio, which represented the maturation of autophasosomes to autolysosomes (Supplementary Fig. 2). Pharmacological perturbation using lysosomal inhibitor chloroquine (CQ) produced synergistic autophagosome accumulation while diminishing mRFP-LC3 positive puncta (Fig. 2, Fig. 2). Immunoblotting analysis confirmed increased endogenous LC3B-II coupled with decreased autophagic substrate p62 levels in HQ-treated group, whereas CQ monotherapy elevated both LC3B-II and p62. Intriguingly, HQ co-treatment partially rescued CQ-induced autophagic blockade (Fig. 2, Fig. 2). These data establish HQ as a potent inducer of autophagic flux *in vitro*.

### HQ protects against alcohol-induced pancreatic injuries by restoring TFEB activities

**Fig. 3:**
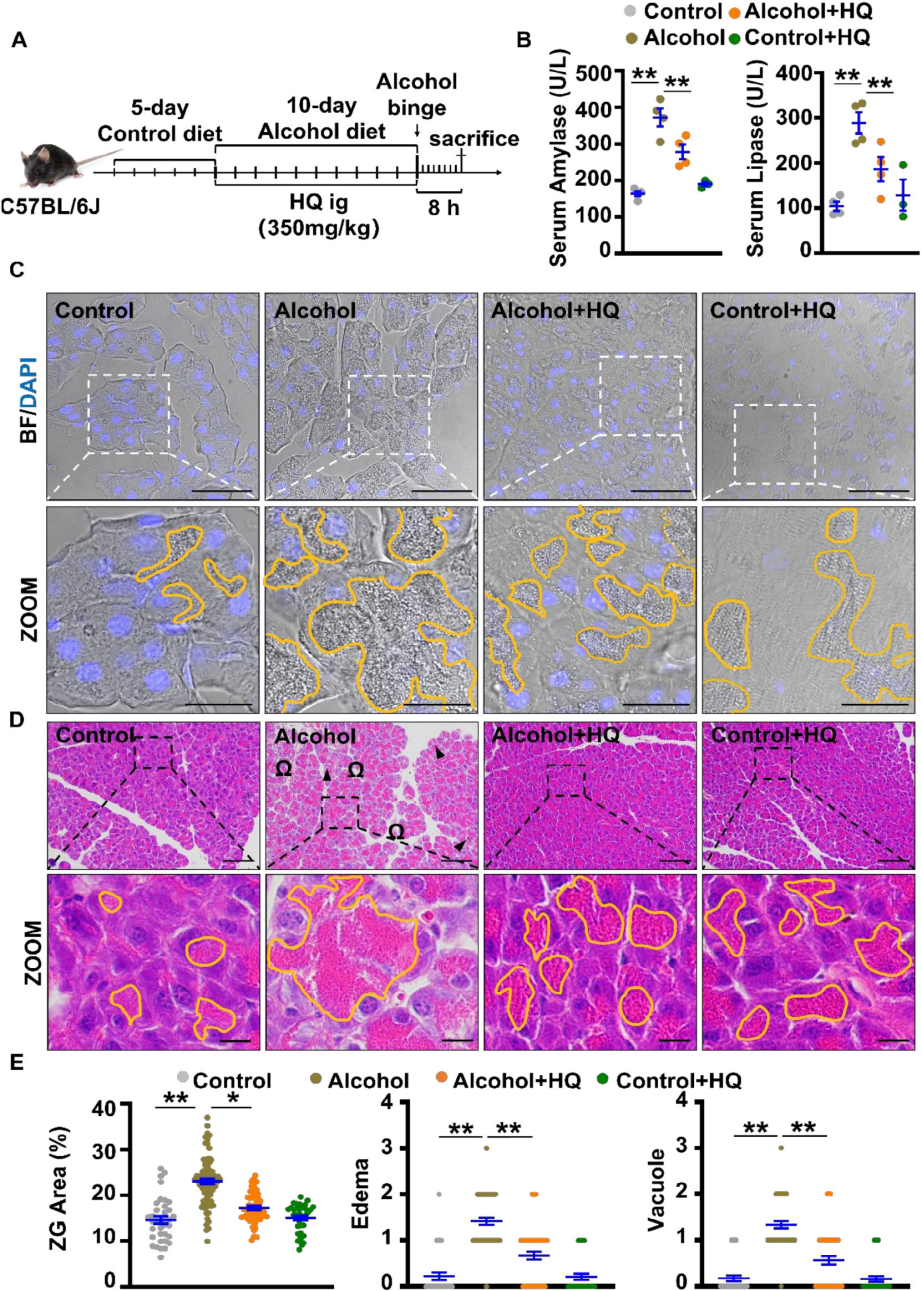
HQ mitigates alcohol-induced pancreatic injuries in mice. (A) Experimental timeline of Gao-binge alcohol feeding model with HQ intervention (350 mg/kg, ig). (B) Serum amylase and lipase were measured. Data are mean ± SEM (n = 3–4). **p < 0.01, *p < 0.05 by one way ANOVA. (C) ZG accumulation was analyzed using cryo-pancreatic tissues. Nuclei were stained with DAPI. Boxed areas were enlarged and shown in the lower panel. Orange circles indicate area of ZG. BF: bright field. Bar: 100 μm. (D) Representative H&E staining images of the C57BL/6J mouse pancreas from alcohol and HQ administration. Omega shows edema and arrow head denotes vacuoles. (E) Individual histology scores of H&E staining were graded. Data are the mean ± SEM (n = 3–4). More than 36 fields were counted in each group. **p < 0.01, *p < 0.05 by one way ANOVA. (For interpretation of the references to colour in this figure legend, the reader is referred to the web version of this article.)

Our previous study revealed that Gao-binge alcohol exposure impairs TFEB-mediated lysosomal biogenesis in mouse pancreas, resulting in insufficient autophagy and pathological ZG accumulation [18]. To validate HQ’s *in vivo* TFEB-activating efficacy, C57BL/6J mice received daily HQ administration concomitant with 10-day chronic alcohol feeding, with final HQ treatment preceding a binge of ethanol (Fig. 3A). HQ intervention significantly attenuated alcohol-induced pancreatic injury biomarkers, reducing serum amylase and lipase levels (Fig. 3B). Histopathological analysis demonstrated alchol-induced ZG accumulation, which was markedly reversed by HQ (Fig. 3, Fig. 3). Pathological scoring confirmed HQ-mediated mitigation of alcohol-induced ZG accumulation, acinar vacuolization and pancreatic edema (Fig. 3E).

**Fig. 4:**
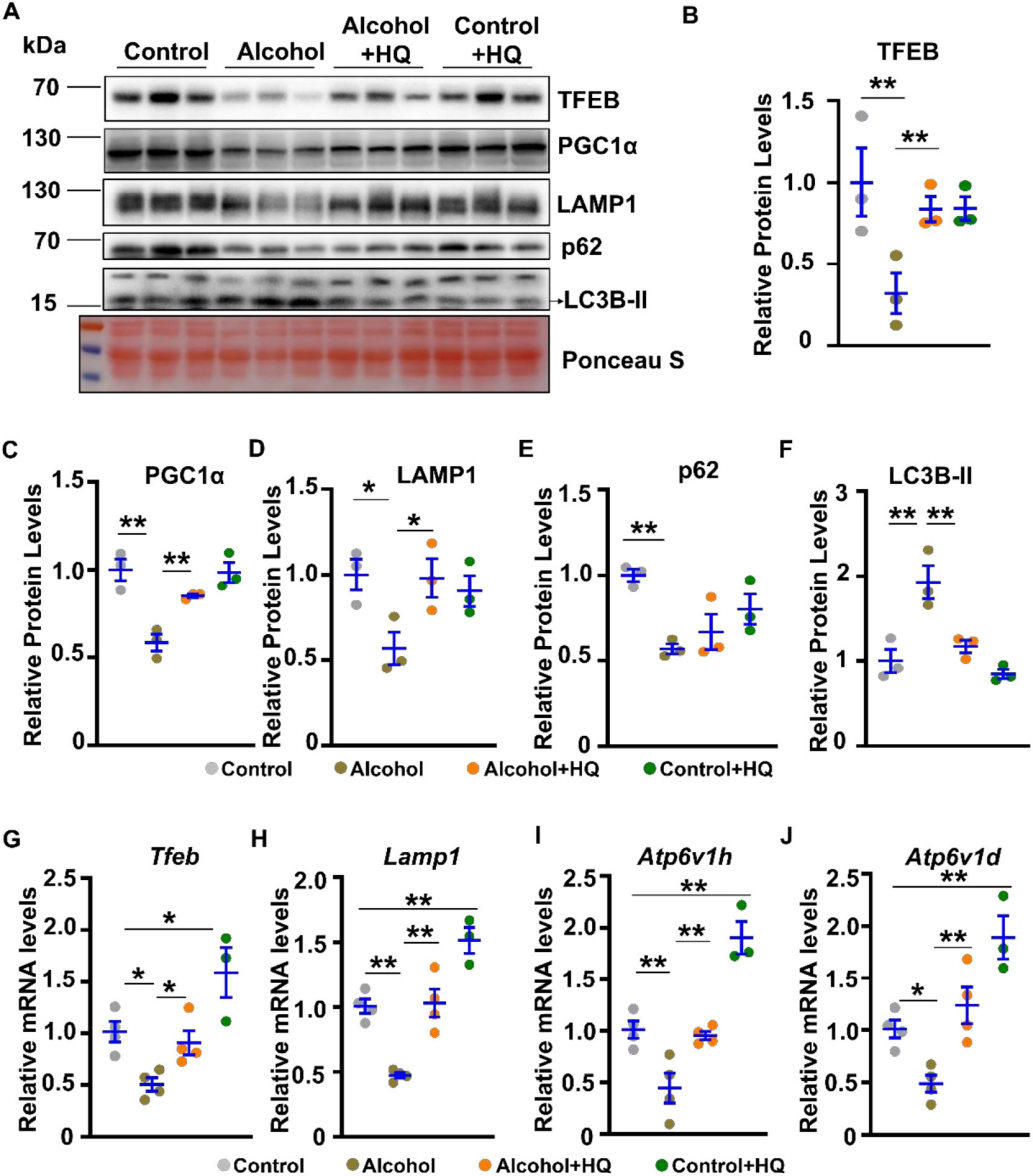
HQ restores TFEB activity in alcoholic pancreatitis mice. (A) Total pancreatic lysates from C57BL/6J mice were subjected to immunoblot analysis. Densitometry analysis was performed for (B) TFEB, (C) PGC1α, (D) LAMP1, (E) p62, and (F) LC3B-II, data are mean ± SEM (n = 3). **p < 0.01, *p < 0.05 by one way ANOVA. (G–J) Pancreatic RNA was extracted for qPCR analysis. The results were normalized to *Rpl13a* and expressed as the multiple change compared to the control group. Data are mean ± SEM (n = 3–4). **p < 0.01, *p < 0.05 by one way ANOVA.

To delineate the TFEB-dependent mechanism, pancreatic lysates underwent molecular characterization. Immunoblot quantification revealed alcohol-induced TFEB depletion and suppression of its downstream targets PGC1α and LAMP1. HQ treatment significantly restored TFEB expression and lysosomal marker LAMP1 levels, thereby effectively rescuing alcohol-induced insufficient autophagy as evidenced by elevated LC3B-II turnover and accelerated p62 degradation (Fig. 4A–F). qRT-PCR analysis demonstrated HQ-induced transcriptional recovery of *Tfeb* mRNA and its downstream lysosomal targets *Lamp1*, *Atp6v1h*, *Atp6v1d* (Fig. 4G–J). These findings conclusively establish HQ-mediated protection against alcoholic pancreatitis through TFEB-driven restoration of lysosomal-autophagic homeostasis.

### TFEB is essential for HQ to exert its protective effects against alcohol-induced pancreatitis

**Fig. 5:**
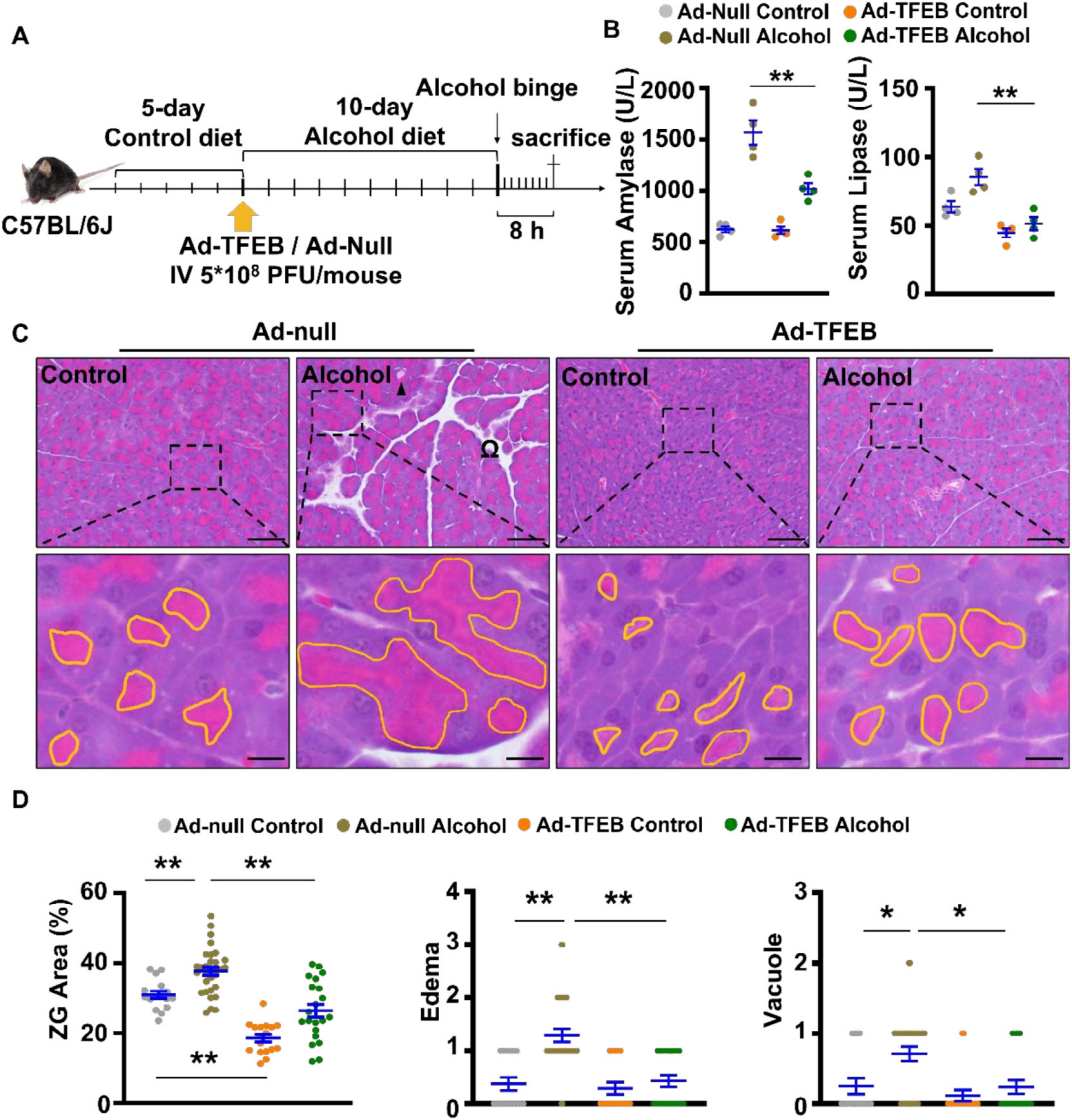
TFEB overexpression ameliorated alcoholic pancreatitis pathology. (A) Experimental timeline of Gao-binge alcohol feeding model with Ad-TFEB overexpression. (B) Serum amylase and lipase were measured. Data are mean ± SEM (n = 4). **p < 0.01 by one way ANOVA. (C) Representative H&E staining images of the C57BL/6J mouse pancreas from alcohol and Ad-TFEB treated groups. Omega shows edema and arrow head denotes vacuoles. Boxed areas were enlarged and shown in the lower panel. Orange circles indicate ZG area. Bar: 100 μm. (D) Individual histology scores of H&E staining were graded. Data are the mean ± SEM (n = 4). More than 16 fields were counted in each group. **p < 0.01, *p < 0.05 by one way ANOVA. (For interpretation of the references to colour in this figure legend, the reader is referred to the web version of this article.)

Given HQ’s protective effects through TFEB activation, we established an adenovirus-mediated pancreatic TFEB overexpression model prior to Gao-binge alcohol exposure to validate its mechanistic centrality (Fig. 5A). Similar to the HQ-treated treatment, TFEB overexpression significantly alleviated alcohol-induced pancreatic injuries as evidenced by reduced serum amylase and lipase levels (Fig. 5B). Histopathological quantification demonstrated markedly suppressed ZG accumulation, acinar vacuolization and pancreatic edema (Fig. 5, Fig. 5).

**Fig. 6:**
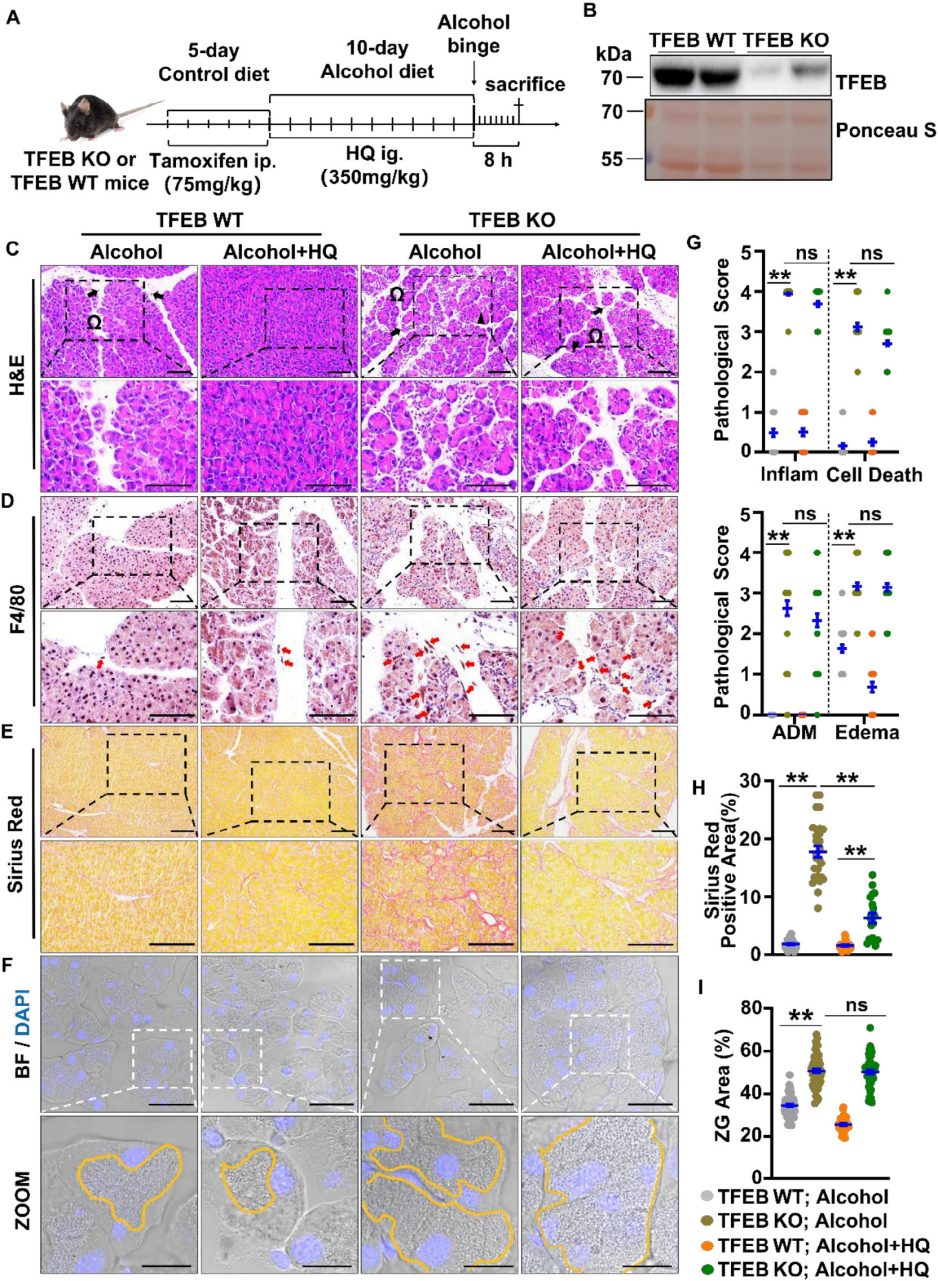
TFEB ablation abolishes HQ-mediated protection in alcoholic pancreatitis. (A) Experimental timeline of Gao-binge alcohol feeding model in TFEB KO mice. (B) Immunoblot analysis of TFEB deletion efficiency in TFEB KO mice. (C) Representative H&E staining images of mouse pancreas from TFEB WT and KO groups treated with alcohol ± HQ. Omega shows edema, arrow head denotes vacuoles and arrow presents inflammatory cells. Boxed areas were enlarged and shown in the lower panel. Bar: 100 μm. (D–E) Representative images of F4/80 and Sirius Red staining are shown. Red arrow indicates positive staining. Boxed areas were enlarged and shown in the lower panel. Bar: 100 µm. (F) ZG accumulation was analyzed using cryo-pancreatic tissues. Nuclei were stained with DAPI. Boxed areas were enlarged and shown in the lower panel. Orange circles indicate area of zymogen granules (ZGs).BF: bright field. Bar: 100 μm. (G–I) Individual histology scores of H&E staining were graded. Area of Sirius Red and ZG were quantified. Data are the mean ± SEM (n = 3–6). More than 18 fields were counted in each group. **p < 0.01, *p < 0.05 by one way ANOVA. (For interpretation of the references to colour in this figure legend, the reader is referred to the web version of this article.)

To further interrogate TFEB’s necessity, we generated pancreatic acinar cell-specific TFEB knockout (KO) mice followed by Gao-binge alcohol feeding (Fig. 6A). Immunoblot analysis confirmed efficient deletion of TFEB in TFEB KO mice compared to wild-type littermates (Fig. 6B). Consistent with our previous data [18], alcohol-exposed TFEB KO mice exhibited exacerbated pancreatitis phenotypes: amplified inflammatory cell infiltration, massive cell death and elevated acinar-to-ductal metaplasia (ADM), which remained refractory to HQ treatment. Moreover, HQ failed to ameliorate alcohol-induced ZG deposition or modulate pathological scores in KO mice (Fig. 6C–I). These finding suggest that HQ counteracts alcoholic pancreatic injuries in a TFEB-dependent manner.

### HQ activates AMPK to promote TFEB activity

**Fig. 7:**
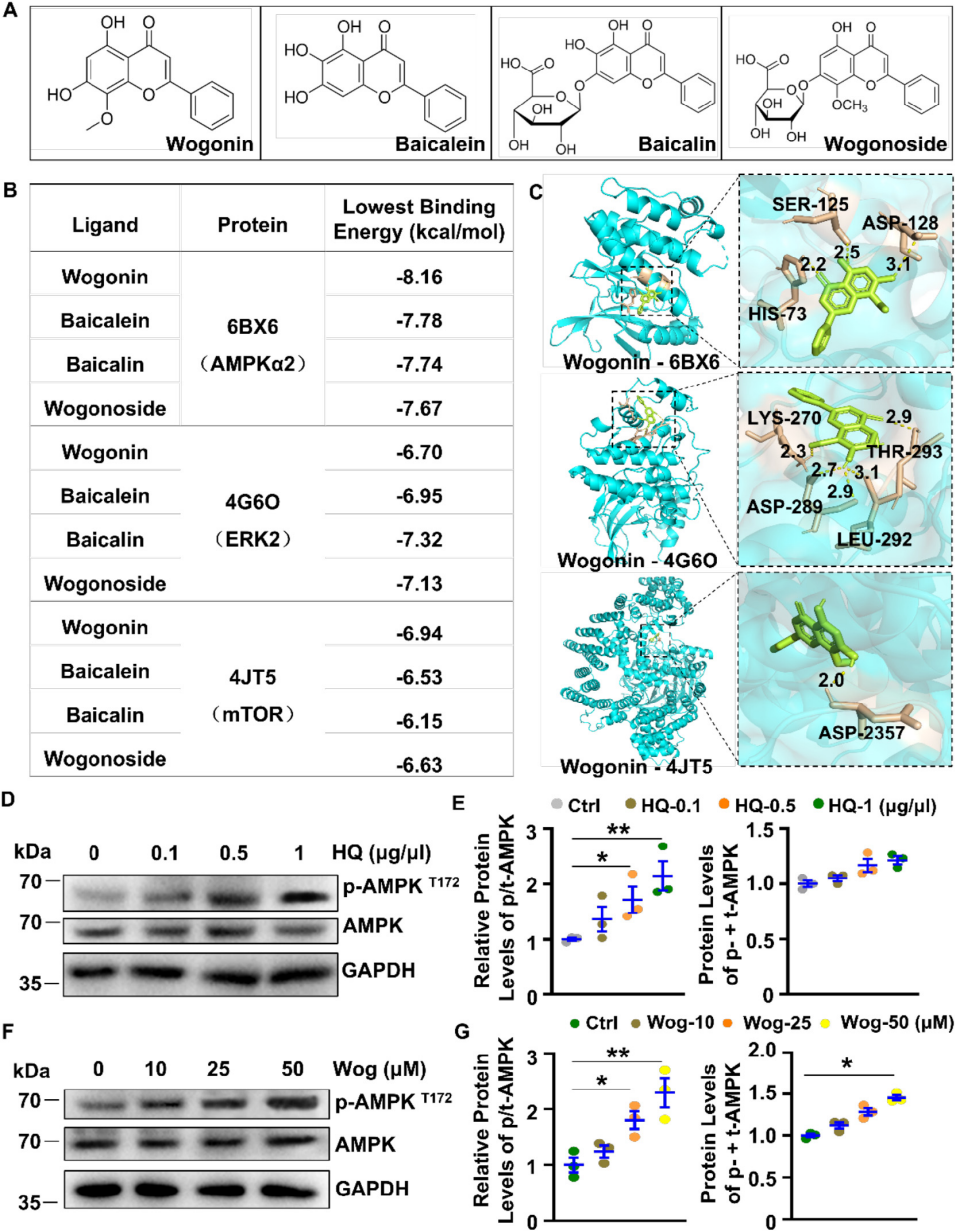
Molecular docking analysis of HQ components with TFEB upstream kinases. (A) Chemical structures of Wogonin, Baicalein, Baicalin, Wogonoside. (B) Optimal binding energies of Wogonin, Baicalein, Baicalin, Wogonoside docking with AMPKα2 (6BX6), ERK2 (4G6O) and mTOR (4JT5), respectively. (C) Optimal binding modes of Wogonin docking with AMPKα2 (6BX6), ERK2 (4G6O) and mTOR (4JT5), respectively. (D) Total cell lysates from HQ-treated cells were subjected to immunoblot analysis. (E) Densitometry analysis was performed for p-AMPK and p-AMPK + t-AMPK combined, data are mean ± SEM (n = 3). **p < 0.01, * p < 0.05 by one way ANOVA. (F) Total cell lysates from Wogonin-treated cells were subjected to immunoblot analysis. (G) Densitometry analysis was performed for p-AMPK and p-AMPK + t-AMPK combined, data are mean ± SEM (n = 3). **p < 0.01, *p < 0.05 by one way ANOVA.

In line with the fact that flavonoids and their glycosides constitute 79.4 % of HQ’s active phytochemical profile [15,19], four flavonoid constituents in HQ ethanol extract-Wogonin, Wogonoside, Baicalin and Baicalein-have been analytically characterized and validated using HPLC-MS techniques (Supplementary Fig. 3). To identify the target(s) of HQ, we performed virtual screening and molecular docking of four major HQ-derived flavonoids (Wogonin, Baicalein, Baicalin and Wogonoside) against TFEB upstream regulators. (Fig. 7A). Molecular docking analysis revealed that AMPKα (6BX6) subunit as the prime target for these flavonoids, among which, Wogonin displayed strongest binding affinity to AMPKα (Fig. 7, Fig. 7). Subsequent key binding site mutagenesis (H73A, S125A or D128A) demonstrated that each mutation reduced Wogonin-AMPK hydrogen bond occupancy and impaired their binding energetics (Supplementary Fig. 4). Previous study showed that AMPK-dependent phosphorylation is required for transcriptional activation of TFEB [8], we validated HQ-AMPK axis *in vitro*. Western blot confirmed HQ dose-dependently induced AMPK phosphorylation at Thr172, a phosphorylation site essential for AMPK activation [20], and upregulated the total AMPK expression (Fig. 7, Fig. 7). Unsurprisingly, Wogonin likewise dose-dependently promoted AMPK expression and phosphorylation at Thr172 (Fig. 7, Fig. 7). These mechanistic insights establish that HQ flavonoids, particularly Wogonin, activate AMPK to potentiate TFEB transcriptional activity.

### Wogonin directly binds to AMPK to alleviate alcohol-induced pancreatic injury and ZG accumulation *in vivo*

**Fig. 8:**
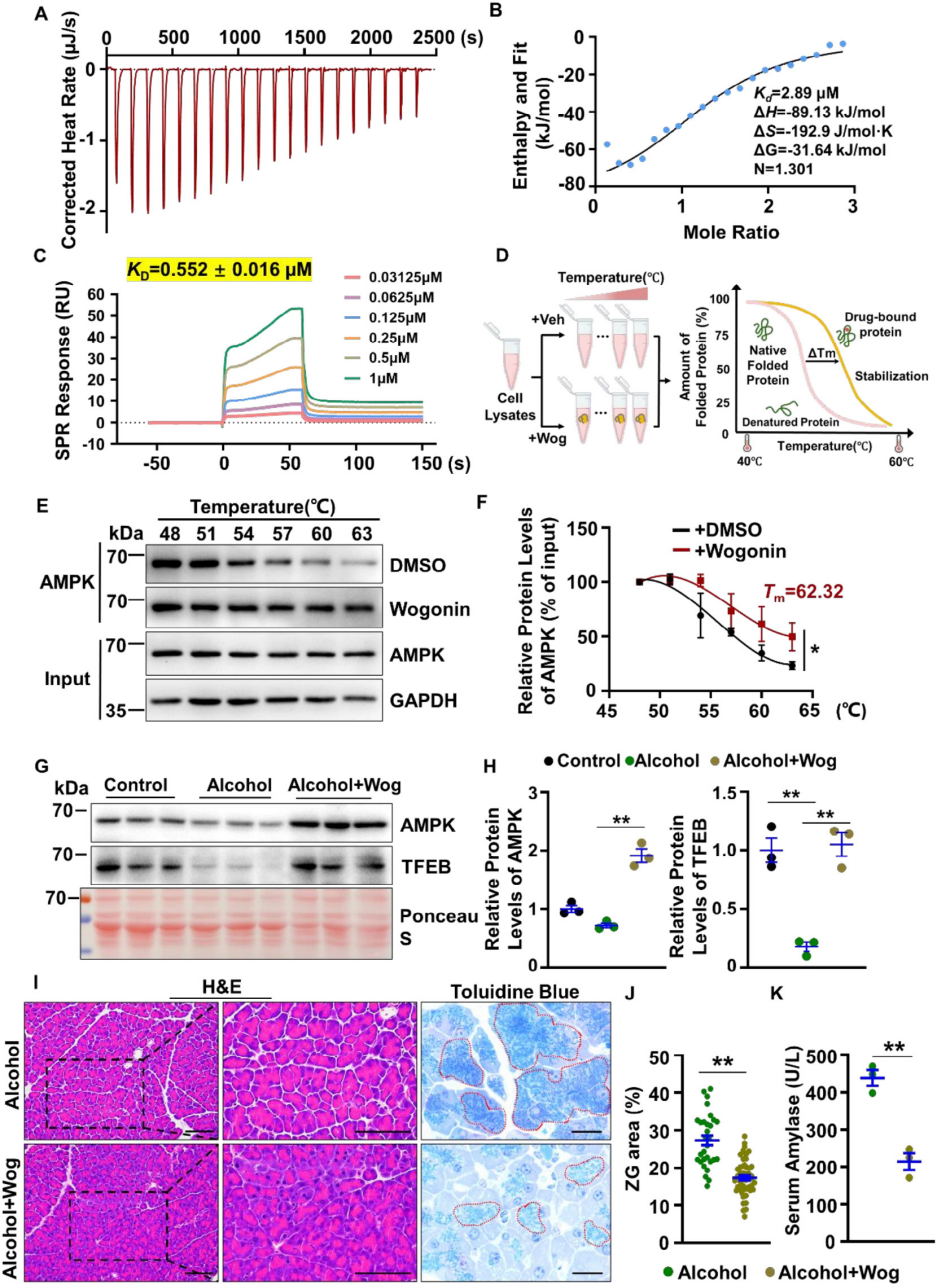
Wogonin directly interacts with AMPK to mitigate alcoholic pancreatitis pathology. (A–B) Isothermal titration calorimetry (ITC) analysis of interactions between Wogonin and AMPK. (C) Surface plasmon resonance (SPR) analysis confirmed high-affinity binding of Wogonin to AMPK. Data are the mean ± SEM (n = 3). (D) Schematic workflow of cellular thermal shift assay (CETSA) for Wogonin-AMPK interaction. (E–F) CETSA-based determination of binding between Wogonin and AMPK. (G) Total pancreatic lysates from Wogonin-treated mice were subjected to immunfoblotting analysis. (H) Densitometry analysis was performed for AMPK and TFEB, data are mean ± SEM (n = 3). **p < 0.01, *p < 0.05 by *t*-test. (I) Representative H&E and Toluidine blue staining images of mouse pancreas from mice treated with alcohol and Wogonin. Boxed areas were enlarged and shown in the right panel. Red circles indicate area of zymogen granules (ZGs). Bar: 100 μm. (J) Area of ZG were counted. Data are the mean ± SEM (n = 3–6). More than 18 fields were counted in each group. **p < 0.01, *p < 0.05 by *t*-test. (K) Serum amylase were measured. Data are mean ± SEM (n = 3). **p < 0.01 by *t*-test. (For interpretation of the references to colour in this figure legend, the reader is referred to the web version of this article.)

To validate the direct Wogonin-AMPK interaction, isothermal titration calorimetry (ITC) was employed to assess the thermodynamic binding parameters. Progressive decreases in binding enthalpy were observed during sequential Wogonin injections into the AMPK solution (Fig. 8A). Thermodynamic analysis revealed the interaction was enthalpically driven (ΔH = −89.13 kJ/mol) with entropic compensation (ΔS = −192.9 J·mol^−1^·K^−1^), demonstrating tight binding characterized by a dissociation constant (Kd = 2.89 μM) (Fig. 8B). Surface plasmon resonance (SPR) analysis, a gold-standard technique for quantifying molecular interactions, further corroborated this binding with improved sensitivity (KD = 0.552 ± 0.028 μM), as evidenced by dose-dependent changes in response units (Fig. 8C & Supplementary Table 1).

Cellular thermal shift assay (CETSA), which exploits ligand-induced thermal stabilization of target proteins, was employed to perform label-free target validation (Fig. 8D). Wogonin-preincubated lysates showed enhanced AMPK thermostability versus DMSO controls. The sigmoidal melting curve shift and average *T_m_* value elevation (57.87˚C to 62.32˚C) confirmed target engagement through conformational stabilization (Fig. 8, Fig. 8). Furthermore, AMPK key binding site mutation (H73A, S125A or D128A) attenuated Wogonin-mediated thermal protection (Supplementary Fig. 5). Collectively, these orthogonal approaches (ITC/SPR/CETSA) establish direct Wogonin-AMPK binding as the mechanism driving conformation-dependent stabilization.

We then examined the *in vivo* AMPK-TFEB activation by Wogonin in alcohol-fed mice. Pharmacokinetic analysis revealed that plasma concentration of Wogonin peaked approximately 30 min after administration, with primary tissue distribution observed in small intestine, liver, pancreas and kidney (Supplementary Fig. 6). In line with previous finding, sub-chronic organ toxicity assay indicated that Wogonin administration induced no detectable pathological lesions or damage in those organs (Supplementary Fig. 7). Pancreatic immunoblotting demonstrated Wogonin restored AMPK and TFEB protein levels versus alcohol-treated controls (Fig. 8, Fig. 8). Histopathological analysis revealed significant mitigation of ZG accumulation (Fig. 8, Fig. 8), paralleled by decreased serum amylase levels (Fig. 8K). Taken together, these data demonstrate that Wogonin protects against AP through direct AMPK targeting, leading to TFEB activation.

### Wogonin activates TFEB *in vitro*

**Fig. 9:**
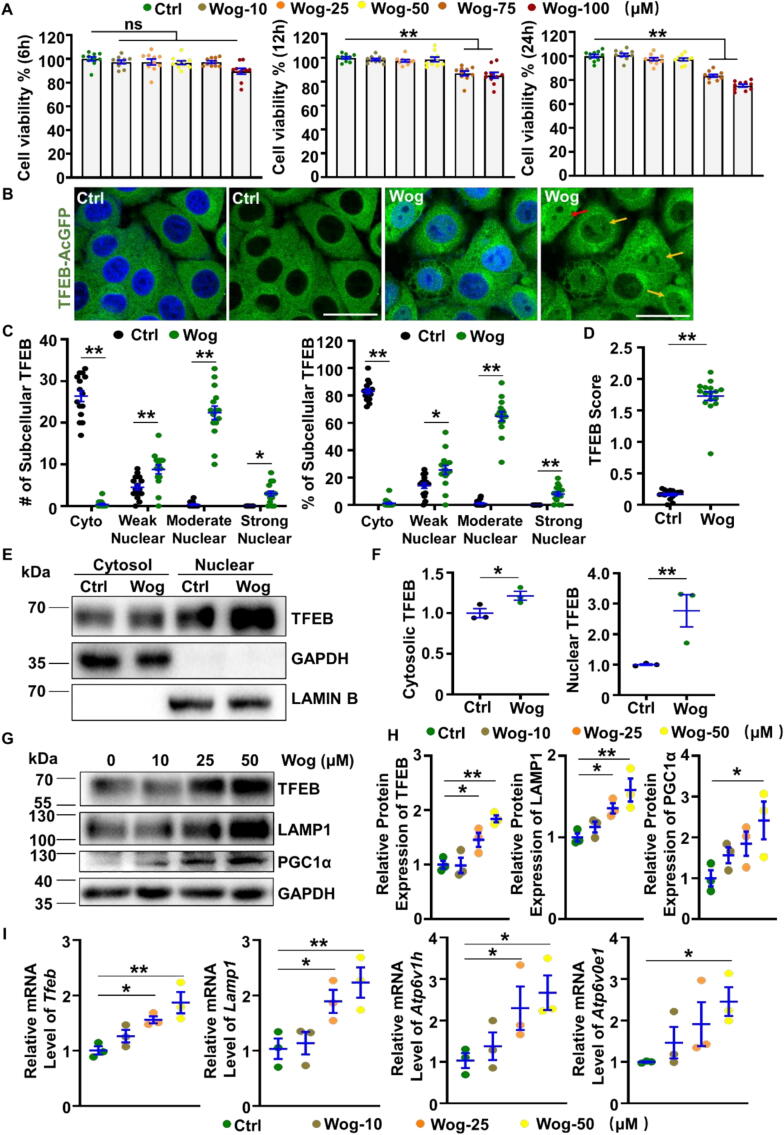
Wogonin activates TFEB *in vitro*. (A) Cell viability was assessed using CCK-8 assay following Wogonin treatment. (B) TFEB distribution was detected in Hela-TFEB-AcGFP expressing cells. Cells were treated with Wogonin (50 μM) for 6 h. Yellow or red arrow denotes moderate or strong nuclear TFEB, respectively. Scale bar = 50 μm. (C) The number and percentage of cytosolic and nuclear TFEB were quantified, data shown are mean ± SEM (n = 3). More than 479 cells were counted in each group. **p < 0.01, *p < 0.05 by Student *t* test. Yellow arrow: weak nuclear TFEB staining, red arrow: moderate nuclear TFEB staining. (D) TFEB score was calculated based on the subcellular distribution of TFEB. (E) Immunoblotting analysis using cytoplasmic and nuclear fractions from Hela cells treated with Wogonin. (F) Densitometry analysis was performed for cytosolic and nuclear TFEB, data shown are mean ± SEM (n = 3). **p < 0.01, *p < 0.05 by Student *t* test. (G) Total cell lysates were subjected to immunoblot analysis. (H) Densitometry analysis was performed for TFEB, LAMP1 and PGC1α. Data shown are mean ± SEM (n = 3). **p < 0.01, *p < 0.05 by one way ANOVA. (I) mRNA was extracted and used for qPCR. Results were normalized to *Gapdh* and expressed as fold change compared to control. Data are mean ± SEM (n = 3). **p < 0.01, *p < 0.05 by one way ANOVA. (For interpretation of the references to colour in this figure legend, the reader is referred to the web version of this article.)

We further verified TFEB activation mediated by Wogonin *in vitro*. The CCK-8 assay demonstrated that Wogonin at ≤50 μg/ml showed no cytotoxicity across all timepoints (Fig. 9A). Substantial nuclear accumulation of TFEB was observed in the stably expressing TFEB-AcGFP Hela cell line after treatment with 50 μM Wogonin for 6 h (Fig. 9B–D). Subcellular fractionation analysis quantification demonstrated that Wogonin promoted TFEB nuclear translocation while elevating endogenous TFEB expression in both subcellular compartments (Fig. 9AE–F). Consistent with the data in Hela cells, we found Wogonin induced TFEB nuclear translocation in both AR42J and 266–6 cells (Supplementary Fig. 8 & 9). Molecular analyses further showed that Wogonin upregulated both TFEB protein and mRNA levels, with coordinated elevation of its transcriptional targets (Fig. 9G–I). These data establish Wogonin as a potent TFEB activator driving transcriptional reprogramming.

### Wogonin promotes autophagic flux via TFEB activation *in vitro*

**Fig. 10:**
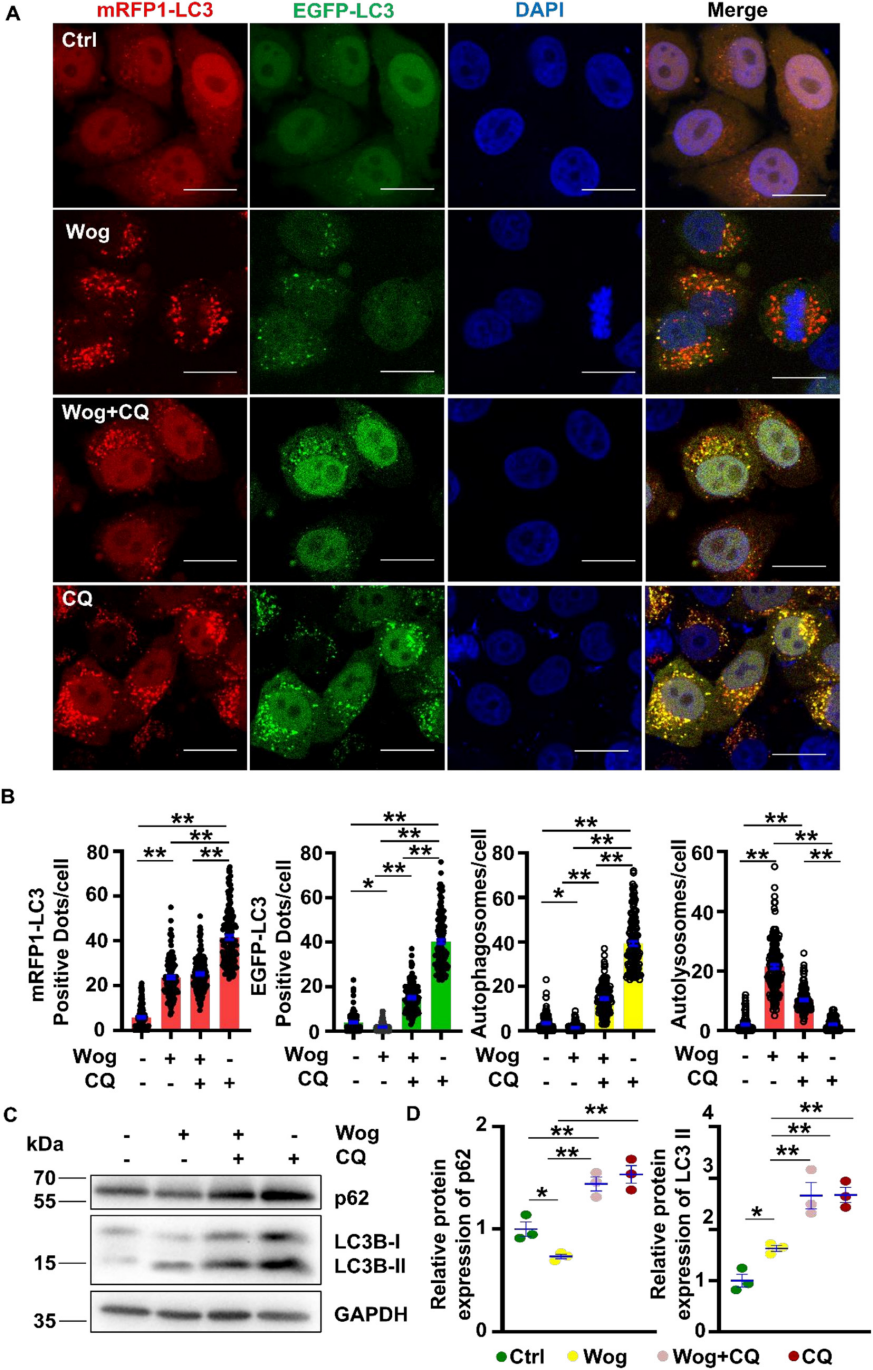
Wogonin promotes autophagy *in vitro*. (A) Hela-EGFP-mRFP-LC3 expressing cells were treated with Wogonin (50 μM) or CQ (25 μM) for 6 h. Representative microscopic images are shown. Scale bar = 20 μm. (B) The number of mRFP1-LC3, EGFP-LC3, autophagosome and autolysosome puncta were quantified. Data are mean ± SEM (n = 3). **p < 0.01, *p < 0.05 by one way ANOVA. 150 cells were counted in each group. (C) Total cell lysates were subjected to immunoblotting analysis. (D) Densitometry analysis was performed for p62 and LC3BII (normalized to loading control GAPDH). Data are mean ± SEM (n = 3). **p < 0.01, *p < 0.05 by one way ANOVA.

To establish TFEB-dependent autophagy modulation, Wogonin was assessed using dual-reporter system. Wogonin treatment increased autophagic progression markers, especially the mRFP-LC3/autolysosome positive puncta. CQ monotherapy increased total autophagosomes but suppressed autolysosomes, while Wogonin co-treatment mitigated inhibition (Fig. 10, Fig. 10). Immunoblotting confirmed flux enhancement: Wogonin increased LC3B-II with p62 clearance, partially reversing CQ-induced LC3B-II and p62 accumulation (Fig. 10, Fig. 10). These synergistic effects conclusively demonstrate Wogonin's TFEB-mediated autophagy activation.

## Discussion

**Fig. 11:**
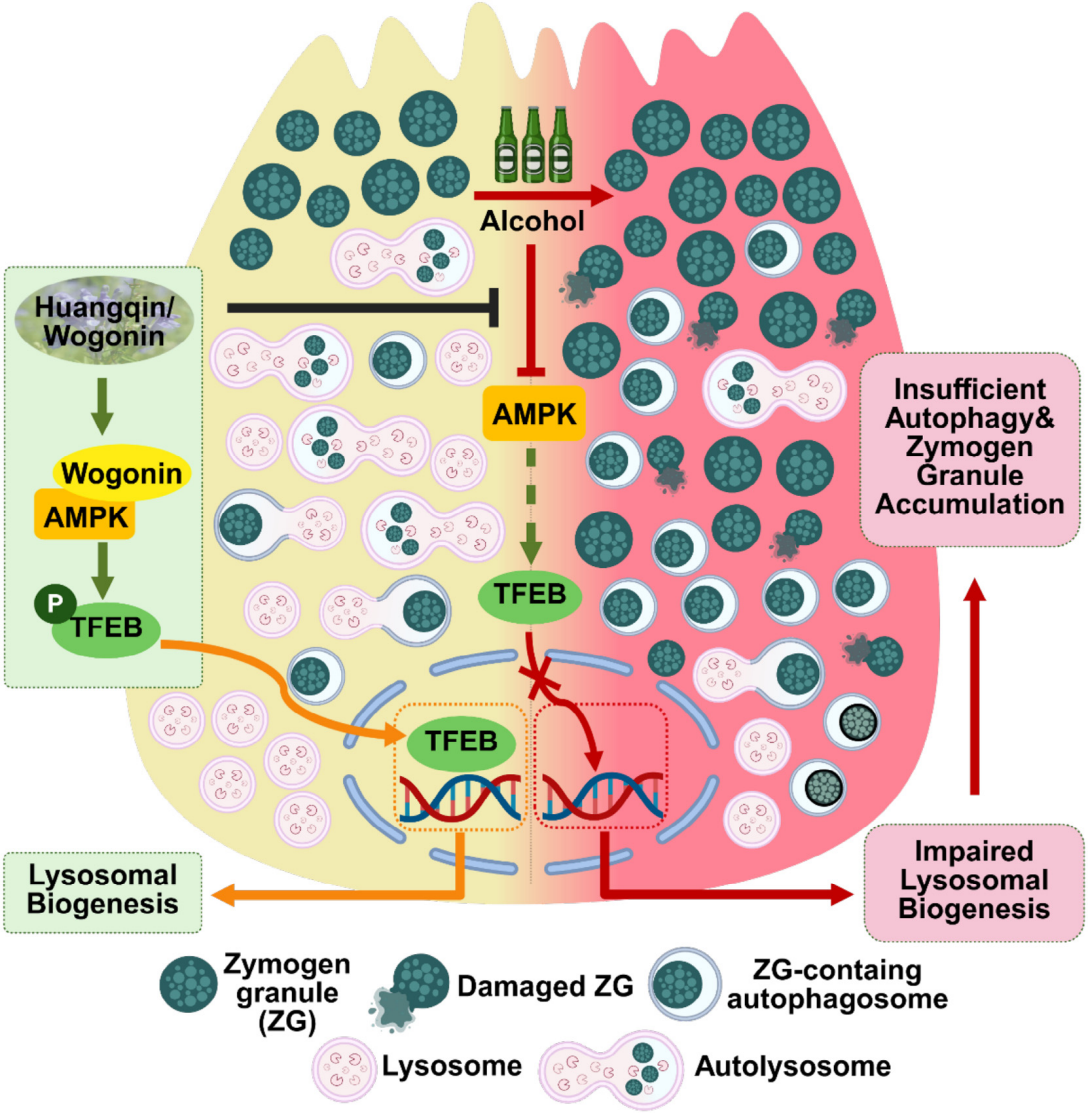
Schematic summary of Wogonin in the treatment of alcohol-induced ZG accumulation and pancreatitis through the AMPK-TFEB pathway. Alcohol consumption decreases AMPK expression, leading to TFEB inactivation and insufficient autophagy. Wogonin directly interacts with AMPK to stabilize its expression to restore AMPK-TFEB signaling, thereby rescuing autophagy flux and attenuating alcohol-induced ZG accumulation and pancreas injuries. Created in BioRender. wang, s. (2025) https://BioRender.com/qa95jbv.

Studies from our group and others have established that impaired TFEB-mediated lysosomal biogenesis and autophagy contribute to both clinical and experimental pancreatitis [4,18,21]. In the present study, we demonstrated that HQ and its key component Wogonin increased TFEB nuclear translocation and transcriptional activity, thereby improving autophagic degradation. Genetic loss-of and gain-of function approaches for pancreatic TFEB confirmed that HQ protected against Gao-binge alcohol-induced pancreatitis in a TFEB dependent manner. Mechanistically, Wogonin directly interacted with AMPKα2, thus activating AMPK and promoting TFEB nuclear translocation and activation. Our findings suggest Wogonin serves as a potential therapy for AP via activating AMPK-TFEB axis (Fig. 11).

Alcohol abuse has long been recognized as a major risk factor for both acute and chronic pancreatitis [22,23]. Chronic alcohol consumption elevates digestive enzyme levels and destabilizes the membrane of ZGs within pancreatic acinar cells, leading to ZG accumulation and the premature enzyme activation [24]. Despite this, only a relatively small proportion of individuals with long-term alcohol use develop pancreatitis, suggesting the presence of intracellular adaptive mechanisms that may mitigate the effects of alcohol. Autophagy, a critical degradation pathway involving the autophagosome-lysosome system, is believed to play a role in these adaptive responses in the pancreas. Indeed, genetic deletion of autophagosome-related genes (ATG5, ATG7, VMP1) or lysosome-related genes (LAMP2, Syntaxin17) compromises autophagy and induces spontaneous pancreatitis [17,[25], [26], [27], [28]]. Additionally, we have previously reported that alcohol impairs TFEB-mediated lysosomal biogenesis and triggers insufficient autophagy. TFEB knockout mice develop severe pancreatitis regardless of alcohol feeding [18]. Here, we provide novel evidences that Wogonin promoted autophagy and protected against alcohol-induced pancreatitis via restoring TFEB expression levels and transcriptional activity, which further confirmed that targeting TFEB and autophagy served as a potential therapy of AP.

TFEB is a basic helix-loop-helix leucine zipper transcription factor that regulates lysosomal biogenesis and autophagy by binding to the CLEAR motif in gene promoters [16]. Its cellular distribution and transcriptional activity are regulated by posttranslational modifications, particularly phosphorylation. At least seven kinases-mTORC1, AKT, MAPK1, MAP4K3, GSK3β, PKCβ, and AMPK- have been reported to modulate TFEB phosphorylation and distribution [29,30]. Among these, mTORC1 phosphorylates TFEB at Ser211, Ser142 and Ser122; AKT at Ser467; MAPK1 at Ser142; MAP4K3 at Ser3; GSK3β at Ser138 and Ser134, resulting in cytosolic retention of TFEB. Conversely, PKCβ phosphorylates TFEB at Ser469, Ser467, Ser463 and Ser462 and AMPK at Ser469, Ser467 and Ser466, promoting TFEB stabilization and nuclear translocation [8,16,[31], [32], [33], [34]]. While mTORC1 is the most well-studied and consistent negative regulator of TFEB, TFEB could be inhibited independently of mTORC1 in AP. Our previous work showed that cerulein activates both mTORC1 and MAPK1, while alcohol specifically increases MAPK1 activity, leading to reduced TFEB expression in the pancreas. However, pharmacologically inhibition of mTORC1 with Torin or MAPK1 with SCH772984 only partially rescued TFEB levels [4,18], indicating that a mTORC1- or MAPK1-independent pathway might be involved in cerulein- or alcohol-induced TFEB inhibition in pancreatitis. Unlike MAPK1 directly phosphorylated TFEB at Ser142 and prevented its nuclear translocation, AKT suppressed TFEB both by indirectly activating mTORC1 and directly phosphorylating TFEB at Ser467 [31]. Our previous work demonstrated that no significant changes on the activation of AKT and its substrate GSK3β in cerulein-induced AP in mice [4]. In contrast to mTORC1 inhibits autophagy, AMPK, a crucial energy sensor that regulates cellular metabolism to maintain energy homeostasis, promotes autophagy by directly activating ULK1 through phosphorylation of Ser 317 and Ser 777 [35]. Furthermore, AMPK inhibits mTORC1 activity by phosphorylating TSC2 and RAPTOR [36,37], which are negative regulators of mTORC1, implying that targeting AMPK might be an effective way to activate TFEB and autophagy. Indeed, researchers found buddleoside, a natural flavonoid compound, alleviates nonalcoholic steatohepatitis via activating AMPK, thereby eliciting phosphorylation of RAPTOR and inhibiting the kinase mTORC1, which activates the TFEB-mediated autophagy-lysosomal pathway [38]. Interestingly, in this study we found that activation of the positive kinase AMPK, rather than inhibition of the negative kinase of TFEB, significantly restored TFEB levels and markedly improved alcohol-induced pancreatic injuries. These findings suggest a novel therapeutic strategy to activate TFEB for pancreatitis treatment.

HQ is commonly used in traditional Chinese medicine for treating inflammatory diseases, featured in formulations such as Dachaihu Tang, Qingyi Tang, and Xiaochaihu Tang [[11], [12], [13]]. Over 120 small molecules have been extracted and identified from HQ, with flavonoids being the principal constituents [15]. Flavonoids, a diverse group of polyphenolic compounds found in various plants, are well-documented for their anti-inflammatory effects. For example, Quercetin inhibits NF-κB activation, reducing levels of pro-inflammatory cytokines TNF-α and IL-6 [39]. Baicalein attenuates inflammatory infiltration and pancreatic injury by blocking MAPK, STAT3, and NF-κB pathways [40]. Isoliquiritigenin activates Nrf2/HO-1 pathway to counteract oxidative stress, thereby mitigating pancreatic tissue damage and pancreatitis-associated lung injury [41]. Wogonin suppresses inflammation response and antioxidant stress to protect against liver injury associated with sepsis, hepatitis B virus injection, and acetaminophen overdose [42]. Among these flavonoids, Wogonin exhibited better NO production inhibition activity in LPS-stimulated macrophages [43]. Limited studies have explored the therapeutic effects and underlying mechanism of HQ/Wogonin on AP. Here, we elucidated the protective effects of HQ/Wogonin on alcohol-induced pancreatitis and explored the underlying mechanisms.

To explore the mechanisms via which HQ/Wogonin alleviate alcohol-induced pancreatitis, TFEB-AcGFP and mRFP-GFP-LC3 stable cell lines were employed. HQ and Wogonin promoted TFEB nuclear translocation and autophagy, respectively. To further demonstrate how HQ activates TFEB to promote autophagy and to identify the key bioacitve component of HQ, virtual screening and molecular docking were conducted. Four major flavonoids from HQ, especially Wogonin, exhibited potential binding to the upstream kinases of TFEB. While comparing with Baicalein, Baicalin and Wogonoside, Wogonin displayed strongest binding affinity to AMPKα (−8.16 kcal/mol), which might attribute to the methoxy group at its C8 position, while Baicalein has a hydroxyl group at the C6 position. This difference improves Wogonin’s lipophilicity and membrane permeability, likely increases its binding affinity to AMPKα [15]. Wogonin directly bound to and stabilized AMPKα, leading to AMPK activation, which resulted in TFEB activation. However, it remains unclear whether HQ’s effects against alcohol-induced pancreatitis were due to a single flavonoid or a combination of multiple flavonoids. Future research is needed to further clarify this in pancreas.

In conclusion, our data demonstrate that Wogonin/HQ activates the AMPK-TFEB pathway to restore autophagy, a critical adaptive mechanism to prevent alcohol-induced pancreatic injuries. These findings deepen our understanding of the molecular mechanisms underlying Wogonin's effects in treating pancreatitis and offer new insights into potential therapeutic approaches for managing this condition.

## Materials and methods

### Animal model of pancreatitis

Alcoholic acute pancreatitis was induced in C57BL/6J mice using acute-on-chronic (Gao-Binge) alcohol mouse model [18]. Briefly, 8–10 weeks old male mice were acclimated to Lieber-DeCarli liquid diet (F1259SP; BioServ, Flemington, NJ) for 5 days followed by ethanol (Decon)-containing (5 %, v/v) liquid diet (F1258SP; BioServ) supplemented with maltose dextrin (Bio-Serv) for 10 days. On the morning of the final day, the mice were orally gavaged with either ethanol (5 g/kg) or a calorie- and volume-matched solution of maltose dextrin. For HQ treatment experiment, HQ was administrated from the day of alcohol feeding by gavage (350 mg/kg) daily. The final dosage of HQ was given half an hour before the alcohol gavage on the last day. For TFEB overexpression experiment, 1 dose of Ad-TFEB (5*10^8^ PFU/mouse, intravenous) or Ad-Null (5*10^8^ PFU/mouse, intravenous) was given on the first day of ethanol feeding. Mice were euthanized 8 h after the gavage, blood samples and pancreatic tissues were harvested. All procedures were approved by the Animal Care and Use Committee of Guangzhou University of Chinese Medicine.

### Generation of pancreatic acinar cell-specific TFEB KO mice

Pancreatic acinar cell-specific TFEB knockout mice were generated as described previously [4]. Briefly, TFEB flox/flox mice were crossed with BAC-Ela-CreERT transgenic mice to produce tamoxifen-inducible pancreatic acinar cell-specific TFEB KO mice. Inducible TFEB KO mice were injected with tamoxifen (75 mg/kg, intraperitoneally) for 5 days. Cre-negative littermates received the same tamoxifen treatment and served as control animals. All mice were further subjected to the Gao-binge alcohol model.

### Reagents

HQ was purchased from Kangmei Pharmaceuticals Co Ltd (Guangdong, China); Lipase kit (L7503-40) and Amylase kit (A7564) were from POINTE Scientific (Lawrenceville, America); The antibodies used for this study were: TFEB (Bethyl, A303-673A); P62/SQSTM1 (Abnova, H00008878-M01); LC3B (Sigma Aldrich, L7543); LAMP1 (Developmental Studies Hybridoma Bank, 1D4B & H4A3); AMPK (Cell Signaling Technology, 5832S); p-AMPK^T172^ (Cell Signaling Technology,2535S); PGC1α (Proteintech, 66369-1-AP);HRP-conjugated goat anti-mouse (115-035-062), HRP-conjugated goat anti-rabbit (111-035-045) and HRP-conjugated goat anti-rat (112-035-143) were from Jackson Immuno Research.

### Preparation of ethanol extraction of HQ

G of HQ were first soaked in 500 ml of 75 % ethanol for 30 min. The mixture was then heated for 1 h using the reflux method. After filtering the extract, the residue was heated again with 500 ml of 75 % ethanol for an additional hour using the same method. Finally, the two extracts were combined. The herbal decoction was concentrated to a viscous state using rotary evaporation machine under negative pressure at 45 °C, and then freeze-dried into a powder using a freeze dryer. Based on the mass of the crude drug and the final mass of the freeze-dried powder, the yield of the crude drug was calculated to be 42.5 %. This indicates that 1 g of freeze-dried powder is equivalent to 2.35 g of the crude drug

### Preparation of tissue lysate and cellular fractionation

Frozen pancreatic tissues were sonicated on ice in radio immuno-precipitation assay buffer (Beyotime, P0013B) supplemented with protease inhibitor cocktail (Active Motif, 37491). Lysates were centrifuged for 15 min at 12,500 g and supernatants were collected and stored at –80 ℃. Nuclear and cytoplasmic fractions of pancreas tissue were prepared by using a commercial kit (Active Motif, 40010). Protein (30 μg) was separated by 8 –12 % SDS-PAGE gel before transfer to a PVDF membrane. Membranes were probed using appropriate antibodies and developed with Chemistar^TM^ high-signal ECL Western Blot substrate (Tanon; 180-5001).

### RNA isolation and real-time quantitative polymerase chain reaction

RNA was isolated from mouse pancreas using RNAex Pro (Accurate Biology; AG21102) and was reverse transcribed into complementary DNA using Evo M-MLV RT Mix Kit with gDNA Clean for qPCR (Accurate Biology; AG11728). qPCR was performed using SYBR Green Premix Pro Taq HS qPCR kit (Accurate Biology; AG11718). Primer sequences (5′ to 3′) for primers used in qPCR are: *Atpv1d* (F: GAGCACAGACTGGTCGAAA, R: AGCTGTCAGTTCCTTCGTGG); *Atpv1h* (F: ATGAGTACCGGTTTGCCTGG, R: GACTGAATGCCAGGAGCCAT); *Pgc1α* (F: ATGTGTCGCCTTCTTGCTCT, R: ATCTACTGCCTGGGGACCTT); *Tfeb* (F: CCAGAAGCGAGAGCTCACAGAT, R: TGTGATTGTCTTTCTTCTGCCG); *Lamp1* (F: AGCATACCGGTGTGTGTCAGTG, R: GTTGGGGAAGGTCCATCCTG); Realtime qPCR results were normalized to *Rpl13a* and expressed as fold of control group.

### Confocal microscopy

Images were acquired using a Leica TSC SPE confocal microscope with a 63 objective (LEICA, TSC SP8). Nuclei were counterstained with DAPI (Servicebio, G1012). EGFP-mRFP-LC3 puncta were counted in 150 cells/ group. TFEB-AcGFP subcellular distribution were counted in ≥ 400 cells/group and scored using the 4-tier system: 0 = exclusively cytosolic; 1 = weak nuclear staining; 2 = moderate nuclear; 3 = strong nuclear. The final TFEB-AcGFP score was calculated using the formula: Strong Nuclear% * 3 + Moderate Nuclear% * 2 + Weak Nuclear% *1 + Cytosolic * 0. All counts derived from three independent biological replicates.

### Histology and histopathological evaluations

Paraffin-embedded pancreas sections were stained with hematoxylin and eosin. Images were taken using a Nikon Eclipse Ni microscope (Nikon, Tokyo, Japan). Pathological evaluations were performed by a well-trained pathologist who was blinded to the group assignments. The extent of pancreatic injury was evaluated semi-quantitatively using a validated scoring system with minor modifications [44]. The specific scoring criteria were as follows: Edema, 0 = no edema; 1 = <10 % of field affected; 2 = 10–25 % of field affected; 3 = 25–40 % of field affected; 4 = >40 % of field affected. Inflammatory cell infiltration, 0 = none or < 2 cells/field, 1 = 2–15 cells/field, 2 = 10–30 cells/field, 3 = 30–50 cells/field, 4 = >50 cells/field. Vacuolization, 0 = none or < 2 vacuoles/field, 1 = 2–25 vacuoles/field, 2 = 25–55 vacuoles/field, 3 = 55–100 vacuoles/field, 4 = >100 vacuoles/field. Cell death, 0 = no or < 2 % of the cells/field, 1 = 2–15 % of the cells/field, 2 = 15–30 % of the cells/field, 3 = 30–60 % of the cells/field, 4 = >60 % of the cells/field. Acinar-to-ductal metaplasia, 0 = no acini affected/field, 1 = <2% of acini affected/field, 2 = 2–15 % of acini affected/field, 3 = 15–30 % of the cells affected/field, 4 = >30 % of acini affected/field.

### Molecular docking

The crystal structures of proteins were retrieved from Protein Data Bank (AMPKα: 6BX6; ERK2: 4G6O; mTOR: 4JT5). Docking process: Discovery Studio Client was used to perform dehydration and hydrogenation of proteins. Pyrx-0.8 and AutoDock Vina39 were used for molecular docking, and Pymol software for mapping.

### Cellular thermal shift assay (CETSA)

Wild-type HeLa cells were cultured overnight in 100- mm dishes. Cellular proteins were obtained using Cell Lysis Buffer supplemented with Proteolytic Protease and Phosphatase Inhibitor Cocktail. The protein content was quantified by the BCA Protein Quantification Kit (23225, Thermo, USA). After the addition of drugs (50 μM) or DMSO, the protein mixture was rotated and mixed for 1 h at room temperature (RT). Subsequently, the protein was subjected to the CETSA protocol, being incubated at temperatures ranging from 48 °C to 63 °C with an increment of 3 °C for 3 min to evaluate its thermal stability. Then, the tubes were centrifuged at 14,000 rpm for 10 min at 4 °C to separate the supernatant from the pellet. 21 μL of the supernatant was mixed with 7 μL of loading buffer and then separated on a 10 % SDS − PAGE for immunoblotting analysis of AMPK.

For AMPK point mutant proteins, AMPK mutant plasmids (H73A, S125A, D128A) were generated by site-directed mutagenesis using the QuikChange II method (Suzhou Synbio Technologies Co., Ltd., Suzhou, China) on a pcDNA3.1(+) vector, digested with NheI and BamHI, transformed into BL21 (DE3) cells, and purified via affinity chromatography for analysis. To express AMPK point mutant proteins, Hela cells were transfected with respective mutant plasmids for 48 h before harvesting.

### Surface plasmon resonance (SPR) assay

The interaction between Wogonin and AMPK was evaluated by the surface plasmon resonance (SPR) assay using the Biacore T200 system (GE Healthcare). The purified recombinant human AMPK (HY − P76142, MCE, USA) was immobilized on a carboxymethylated 5th sensor chip via a standard amine coupling method with an amino-coupling kit. Analytes were injected as various concentrations of Wogonin (ranging from 0.03125 μM to 1 μM) in PBS-P running buffer. The data were analyzed and fitted to a 1:1 Langmuir model using the Biacore™ 1 K SPR molecular interaction system, which is designed for robust and reproducible molecular interaction analysis to obtain results.

### Isothermal titration calorimetry (ITC)

Isothermal titration calorimetry (ITC) was carried out with a NANO − ITC instrument (TA) to explore the interaction between AMPK protein and Wogonin. The AMPK protein was dissolved in a PBS solution containing 1 % (v/v) DMSO. Wogonin was diluted in the same buffer as that of the protein. Aliquots (2 μL) of the Wogonin solution, with a concentration 20 times the binding − site concentration, were injected into the reaction cell filled with 0.3 mL of a 0.002 mM AMPK protein solution via a 50 μL rotating stirrer − syringe. Titration interval was 120 s; the temperature was set at 25 °C; and the stirring rate was 350 r/min. In separate titrations of the ligand into the buffer solution, the heat of dilution was determined to be negligible. Calorimetric data were analyzed using NANO − ITC Analysis Software (TA).

### Toluidine blue staining

Small pancreas specimens were fixed in 2.5 % glutaraldehyde and then in 1 % OsO_4_ for 1 h. After dehydration, the specimens were embedded in epoxy resin. Thin sections (1 μm) were cut and stained with 1 % toluidine blue buffer. Images were captured using a Nikon Eclipse Ni microscope (Nikon, Tokyo, Japan).

### Statistical analysis

All experimental data were presented as mean ± SEM. Multiple group comparisons employed One-way ANOVA analysis with Bonferroni post hoc test, while two group comparisons were analyzed using Student *t* test, as applicable. p < 0.05 was considered significant.

## Compliance with ethics requirements

The experiments involving animals were approved by the Animal Care and Use Committee of Guangzhou University of Chinese Medicine (no. 20220101).

## Declaration of competing interest

The authors declare that they have no known competing financial interests or personal relationships that could have appeared to influence the work reported in this paper.
