# Supplementary material for: Wogonin, a bioactive flavonoid from Scutellaria baicalensis, alleviates alcoholic pancreatitis via AMPK-mediated TFEB activation
Source: J Adv Res. 2025 Nov 10;86:891–907. doi: 10.1016/j.jare.2025.11.006 (PMC13453865; doi:10.1016/j.jare.2025.11.006)
Supplement: Supplementary file 1 [file mmc1.pdf]

Wogonin, a bioactive flavonoid from Scutellaria baicalensis, alleviates alcoholic pancreatitis via AMPK-mediated TFEB activation

Supplementary materials and methods

Confocal Microscopy

Lysosomes were immunostained with LAMP1 (Developmental Studies Hybridoma Bank, H4A3). Nuclei were counterstained with DAPI (Servicebio, G1012). Images were acquired using a Leica TSC SPE confocal microscope with a 63 objective (LEICA, TSC SP8).

Plasma pharmacokinetics study

The pharmacokinetic analysis of Wogonin was performed as described previously [1]. Blood samples were collected from the mice prior to intragastric administration of Wogonin as blank controls. The time points of blood sampling were as follows: 5, 10, 20, 30, 60, 120, 180, 240, 360, 540, 600 and 720 min, respectively. A total of 20 μL blood was collected at each time point, and then centrifuged at 3500 × g for 10 min at 4 °C. The samples were precipitated with acetonitrile and centrifuged at 8000 × g for 15 min at 4 °C. The supernatant was detected using the UHPLC-MS/MS analysis, and the pharmacokinetic parameters were calculated using LabSolution software (Shimadzu Corporation, Kyoto, Japan).

UHPLC-MS/MS analysis

UHPLC-MS/MS analysis was performed in both positive and negative ionization modes using an Agilent 1290 ultra-high performance liquid chromatography system, equipped with a 6490 triple quadrupole mass

spectrometer (Agilent, California, USA), respectively. The chromatographic column was ZORBAX SB-AQ C18 column (4.5 ×250 mm, 5 μm). The injection temperature was 40 °C, and the injection volume was 5 μL. The mobile phase consisted of 0.01 % formic acid in water and acetonitrile. The analysis was carried with an elution gradient as follows: 0–1 min,45 % B; 1–3 min, 60 % B; 3–4 min, 95 % B; 4–5 min, 5 % B; the flow rate is 0.4 mL/min.

Mass spectrometric analysis was performed in multiple reaction monitoring (MRM) mode. The total run time was 6.5 minutes. The following ion pairs were monitored in positive ion mode: for baicalin, $[M-H]^-$ at m/z 447.20 → 271.2; for wogonoside, $[M-H]^-$ at m/z 461.25 → 285.2; for baicalein, $[M-H]^-$ at m/z 271.22 → 123.1; and for wogonin, $[M-H]^-$ at m/z 285.20 → 270.2.

Sub-chromic Toxicity Study

To assess the sub-chronic toxicity of wogonin, 8-weeks old healthy male C57BL/6J mice were used. Wogonin was suspended in 0.5% sodium carboxymethyl cellulose (CMC-Na) solution and mice were administered orally at dose of 10 mg/kg daily for 14 days. Control mice were administered 0.5% CMC-Na. Body weight, food, and water intake were recorded daily throughout the study period. On the final day, mice were anesthetized, and blood, liver, pancreas, kidney and small intestine samples was collected. Serum alanine aminotransferase (ALT, U/L), amylase (AMY, U/L), and creatinine (μM) levels were measured with commercial kits following the manufacturer's instructions. Paraffin-embedded sections were stained with hematoxylin and eosin (H&E) for histopathological evaluation.

Tissue distribution study

To evaluate the tissue distribution of wogonin, 8-weeks old healthy male C57BL/6J mice were used. Wogonin was suspended in 0.5% CMC-Na solution and mice were orally administered wogonin at the dose of 50 mg/kg.

Based on its plasma Tmax, blood was collected from the orbital venous plexus, and the liver, pancreas, kidney and small intestine were then collected. Tissues were rinsed with 0.9% saline, blotted dry, and preserved for subsequent analysis. 100 mg of tissue was homogenized in PBS. The homogenate was precipitated with acetonitrile containing 0.2% formic acid and 10 ng/mL nobiletin, followed by centrifugation at 8000 × g for 15 minutes at 4 °C. The supernatant was dried in a vacuum concentrator, reconstituted in 50% acetonitrile, centrifuged again, and the final supernatant was used to quantify wogonin concentrations in each tissue by UHPLC-MS/MS.

Supplementary Figure 1

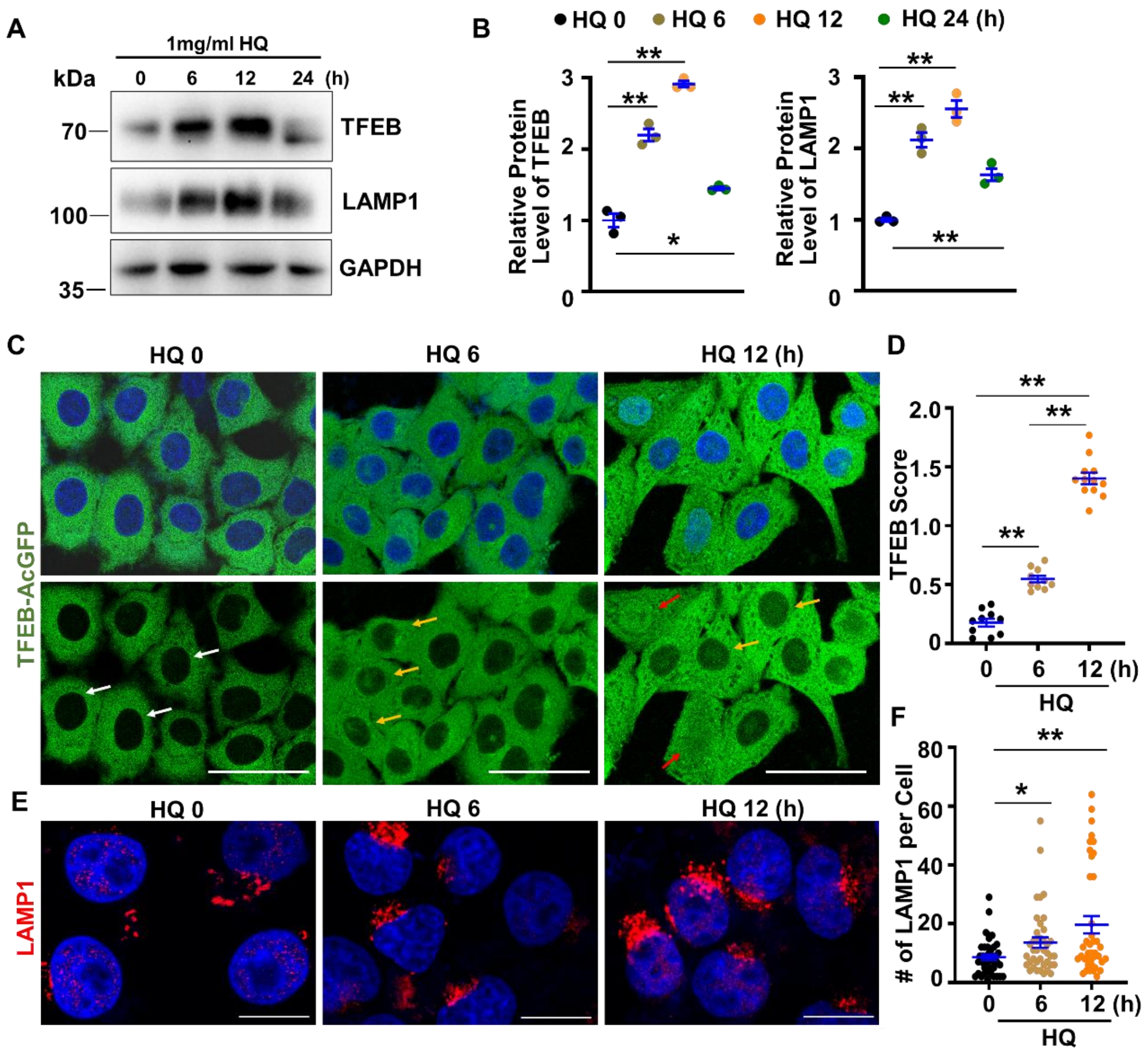

Supplementary Figure 1. HQ activates TFEB in a time-dependent manner within 12h. (A) Immunoblotting analysis using Hela cell lysates treated with 1mg/ml HQ for 0, 6, 12 and 24h, respectively. **(B)** Densitometry analysis was performed for TFEB and LAMP1, data shown are mean ± SEM (n = 3). **p < 0.01, *p < 0.05 by one way ANOVA. **(C)** TFEB nuclear translocation was assessed in TFEB- AcGFP expressing Hela cells after treated with 1mg/ml HQ for 0, 6 and 12h, respectively. White arrow: no nuclear TFEB staining, yellow arrow: moderate nuclear TFEB staining, red arrow: strong nuclear TFEB staining. **(D)** The number and percentage of cytosolic and nuclear TFEB were quantified, data shown are mean ± SEM (n = 3). More than 400 cells were counted in each group. **p < 0.01, *p < 0.05 by one way ANOVA. **(E)** Lysosomes were stained with LAMP1 from Hela cells treated with 1mg/ml

HQ for 0, 6 and 12h, respectively. **(F)** The number of lysosome was counted ,data shown are mean ± SEM (n = 3). Over 40 cells were counted in each group. **p < 0.01 by one way ANOVA.

Supplementary Figure 2

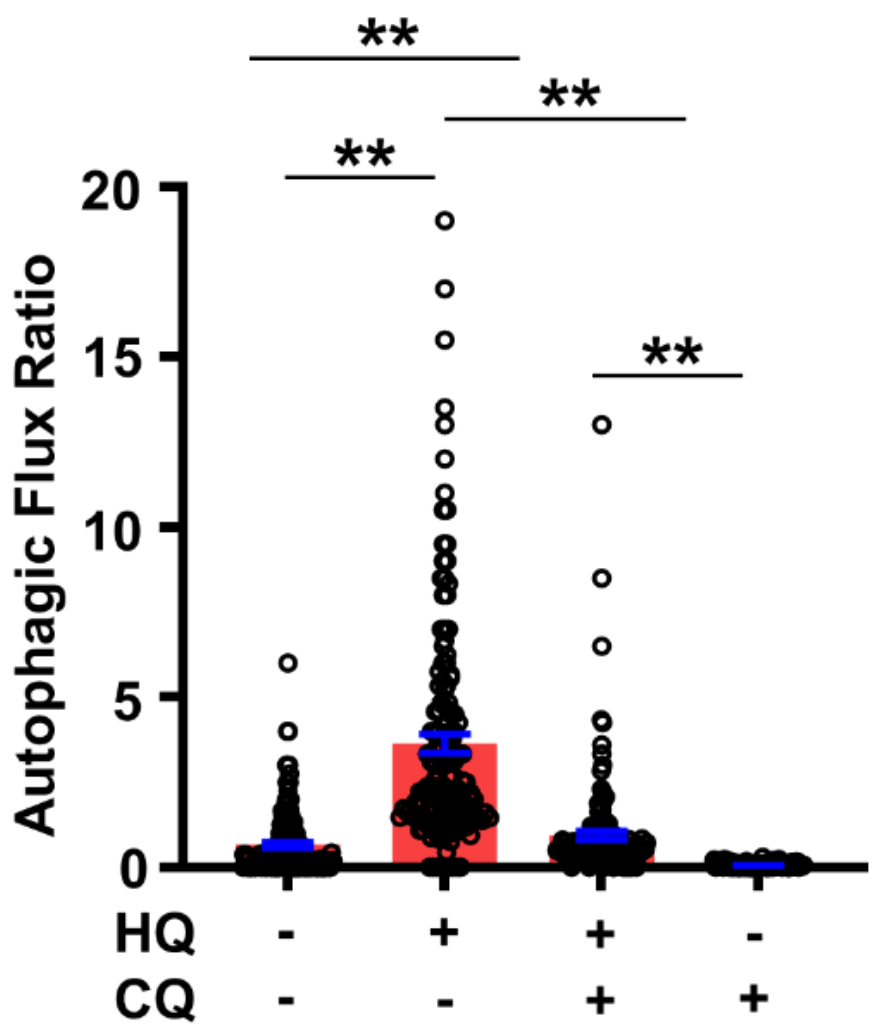

Supplementary Figure 2. HQ treatment increases autophagic flux ratio. Autophagic flux ratio (autophagic flux ratio = number of red-only puncta/number of yellow puncta) was calculated in control, HQ, HQ+CQ and CQ groups. Data shown are mean ± SEM (n = 3). 150 cells were counted in each group. **p < 0.01 by one way ANOVA.

Supplementary Figure 3

A

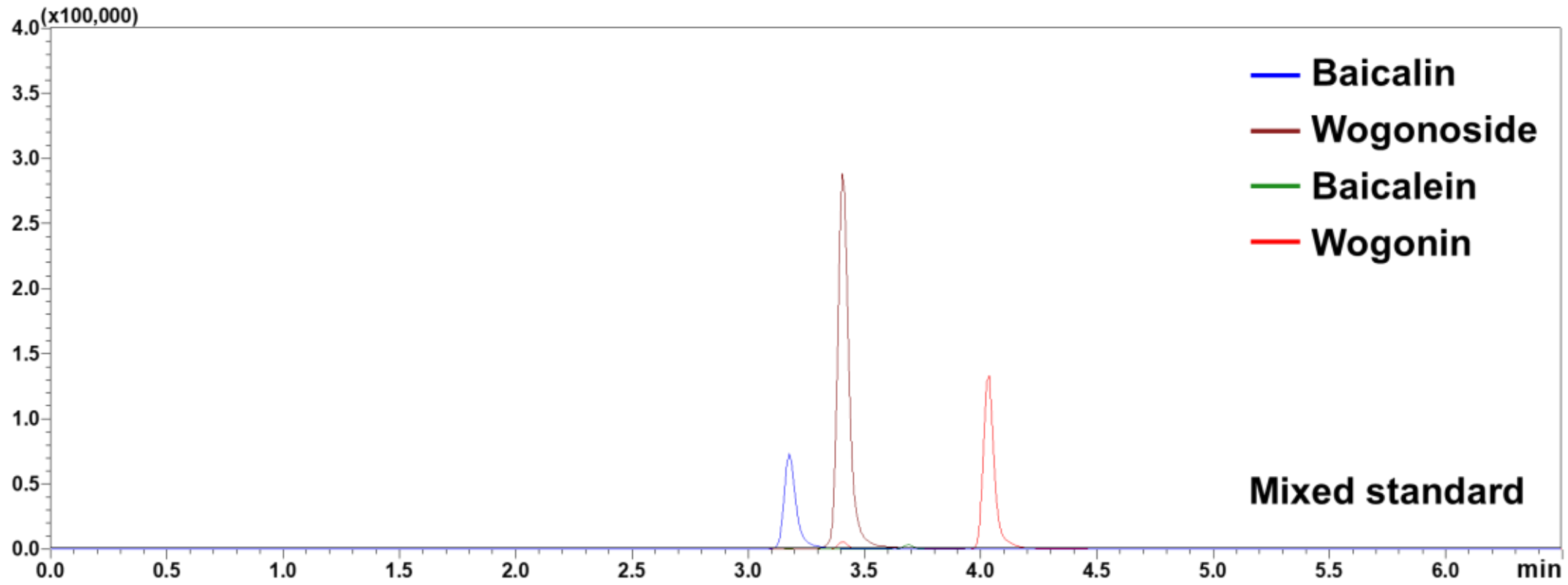

B

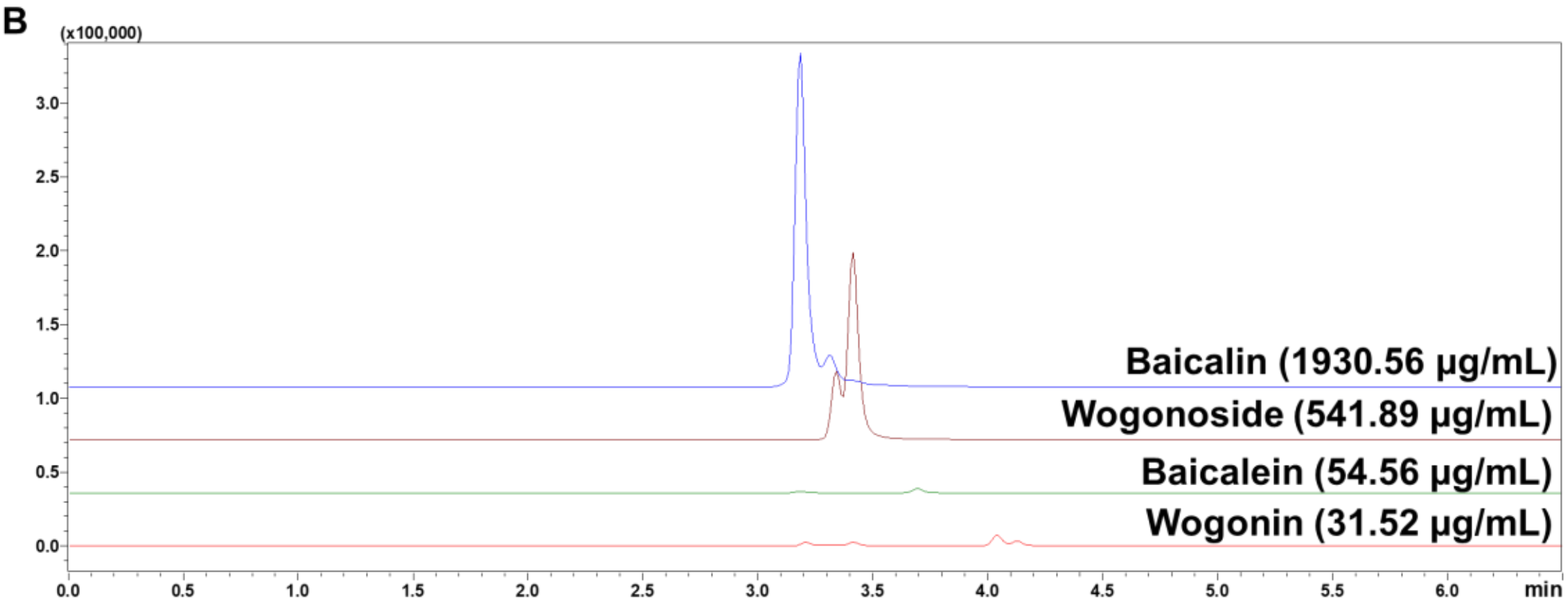

C

Identification	Molecular Formula	Molecular Weight	RT/min
Wogonin	$C_{16}H_{12}O_5$	284.26	4.458
Baicalein	$C_{15}H_{10}O_5$	270.24	4.080
Wogonoside	$C_{22}H_{20}O_{11}$	460.39	3.751
Baicalin	$C_{21}H_{18}O_{11}$	446.36	3.483

Supplementary Figure 3. Component profiling in HQ ethanol extract. (A) HPLC profile of reference standards for main HQ constituents; **(B)** Representative HPLC profile of HQ ethanol extract identifying dominant components; **(C)** Structural validation of main HQ compounds by mass spectrometry.

Supplementary Figure 4

A

Ligand	Protein (6BX6)	Lowest Binding Energy (kcal/mol)
Wogonin	WT	-8.16
	H73A	-7.53
	S125A	-6.68
	D128A	-6.39

B

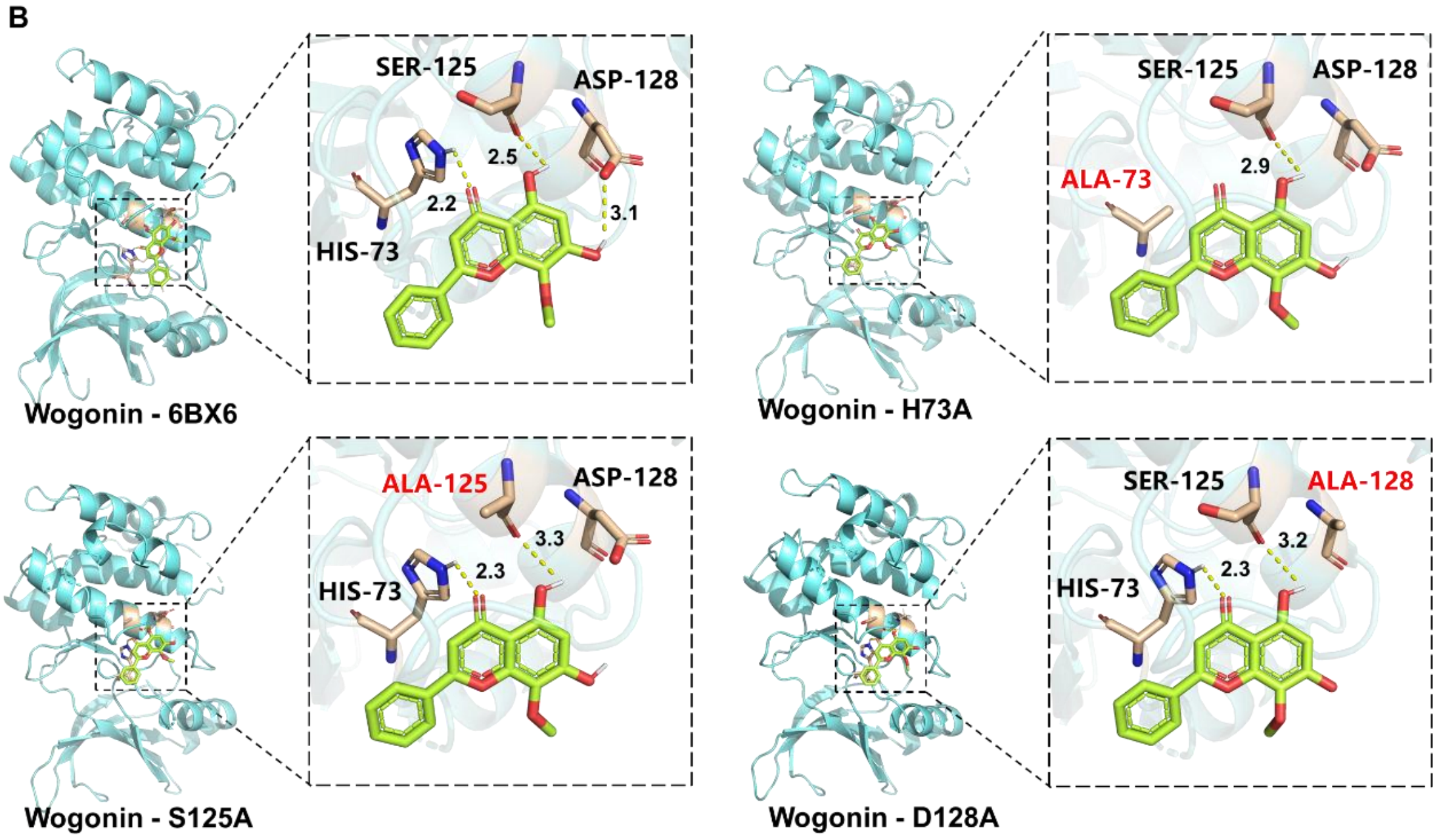

Supplementary Figure 4. Site directed mutations reduce wogonin-AMPK binding affinity. (A) Single-point alanine substitutions at H73, S125, and D128 decrease wogonin-AMPK binding affinity; **(B)** H73A, S125A, and D128A mutations inhibit wogonin-AMPK hydrogen bond formation.

Supplementary Figure 5

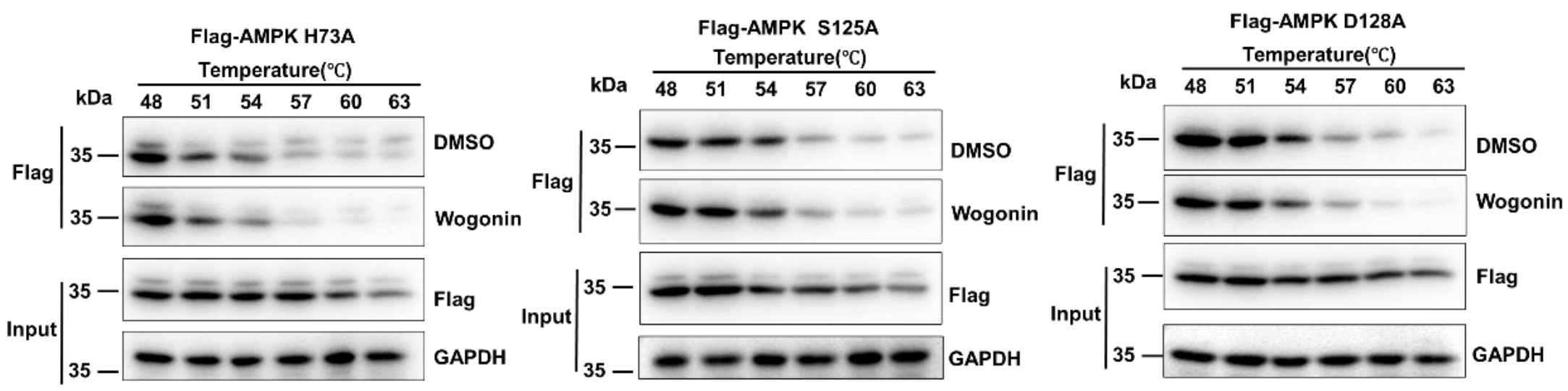

Supplementary Figure 5. AMPK key binding site mutation abolishes wogonin-mediated AMPK stabilization. AMPK (6BX6) single-point alanine substitution at H73, S125, or D128 suppresses wogonin-AMPK binding affinity.

Supplementary Figure 6

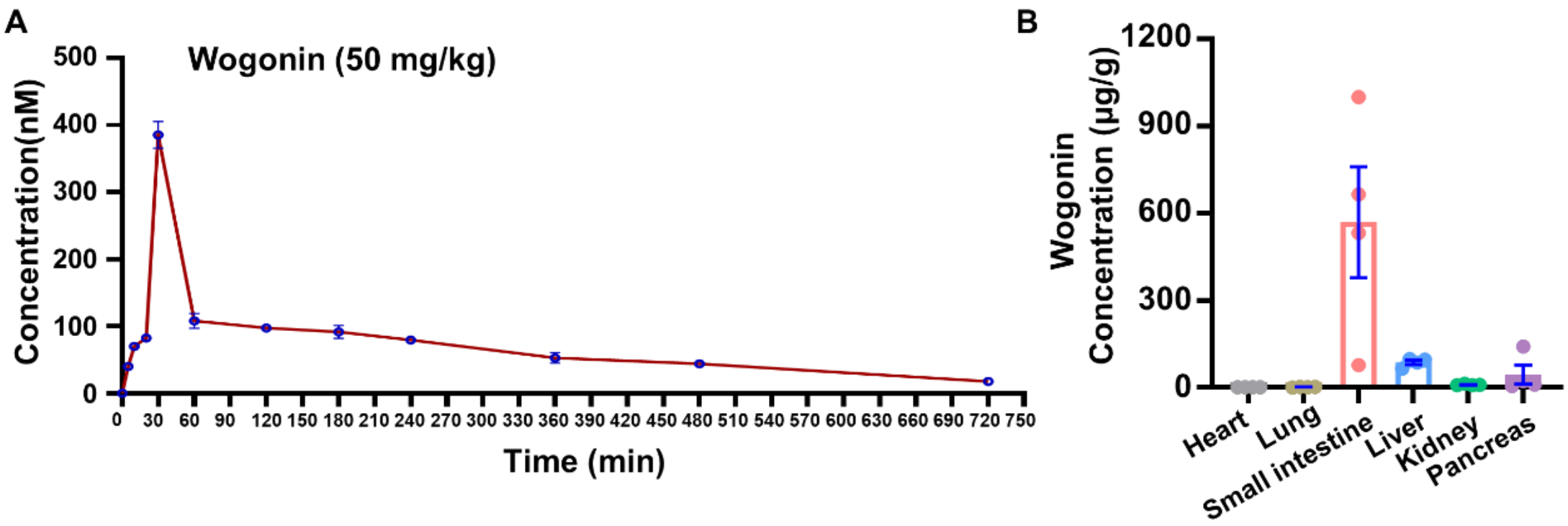

Supplementary Figure 6. Pharmacokinetic analysis of Wogonin. Mice were administered wogonin intragastrically as a single dose of 50 mg/kg. **(A)** Blood samples were collected at 0, 5, 10, 20, 30, 60, 120, 180, 240, 360, 480 and 720 min post-administration, followed by HPLC-MS analysis. Data represent mean ± SEM (n = 6). **(B)** Heart, lung, small intestine, liver, kidney, and pancreas samples were collected 30 min post-administration, followed by HPLC-MS analysis. Data represent mean ± SEM (n = 4).

Supplementary Figure 7

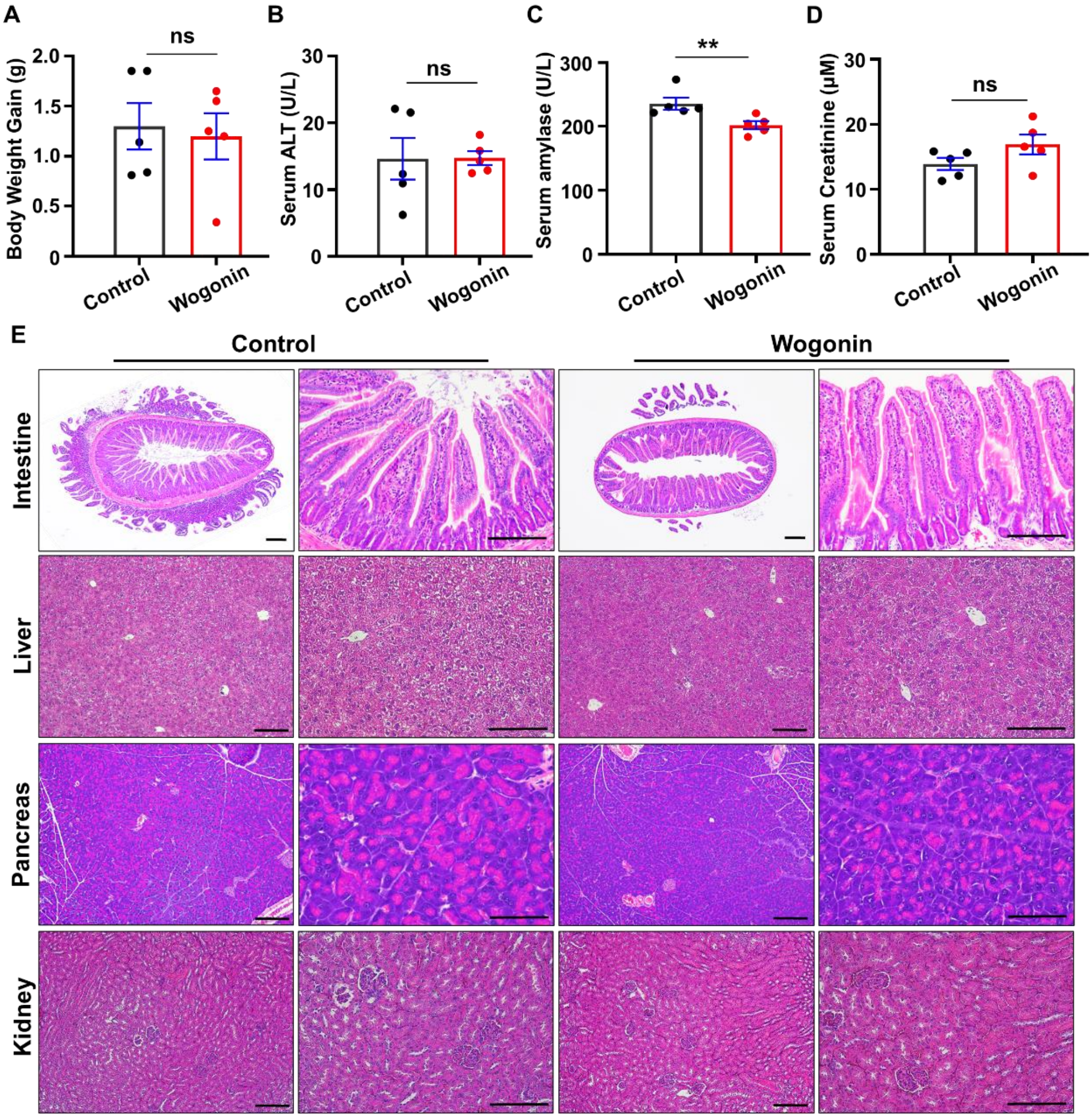

Supplementary Figure 7. Tissue toxicity evaluation of Wogonin. Mice were orally administered Wogonin daily at 10 mg/kg for 14 days; tissue and blood samples were collected on day 14. **(A)** Body weight gain, and serum levels of **(B)** ALT, **(C)** amylase and **(D)** creatinine were measured to evaluate organ toxicity. Data are presented as mean ± SEM (n = 5). **p < 0.01 versus control; Student's t-test. ns: not significant. **(E)** Representative H&E staining images of the small intestine, liver, pancreas and kidney following wogonin administration. Bar: 50 μm.

Supplementary Figure 8

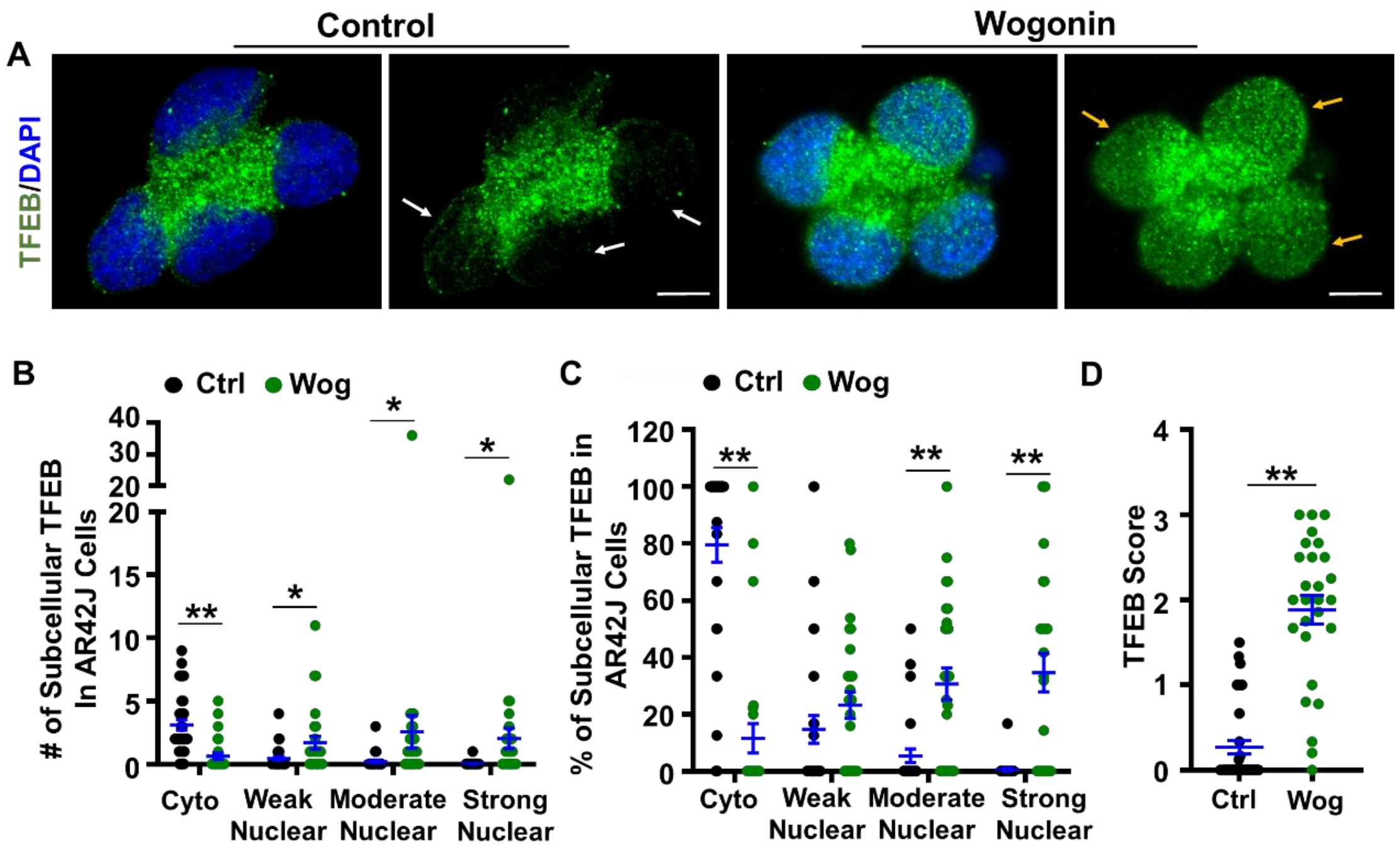

Supplementary Figure 8. Wogonin induces endogenous TFEB nuclear translocation in pancreatic AR42J cells. (A) AR42J cells were treated with 50 μM Wogonin for 6h, then endogenous TFEB was detected with TFEB antibody. White or yellow arrow denotes weak or moderate nuclear TFEB, respectively. Scale bar = 50 μm. **(B)** The number and **(C)** percentage of cytosolic and nuclear TFEB were quantified, data shown are mean ± SEM (n = 3). More than 130 cells were counted in each group. $^{**}p < 0.01$, $^{*}p < 0.05$ by Student t test. **(D)** TFEB score was calculated based on the subcellular distribution of TFEB. Data shown are mean ± SEM (n = 3). $^{**}p < 0.01$ by Student t test.

Supplementary Figure 9

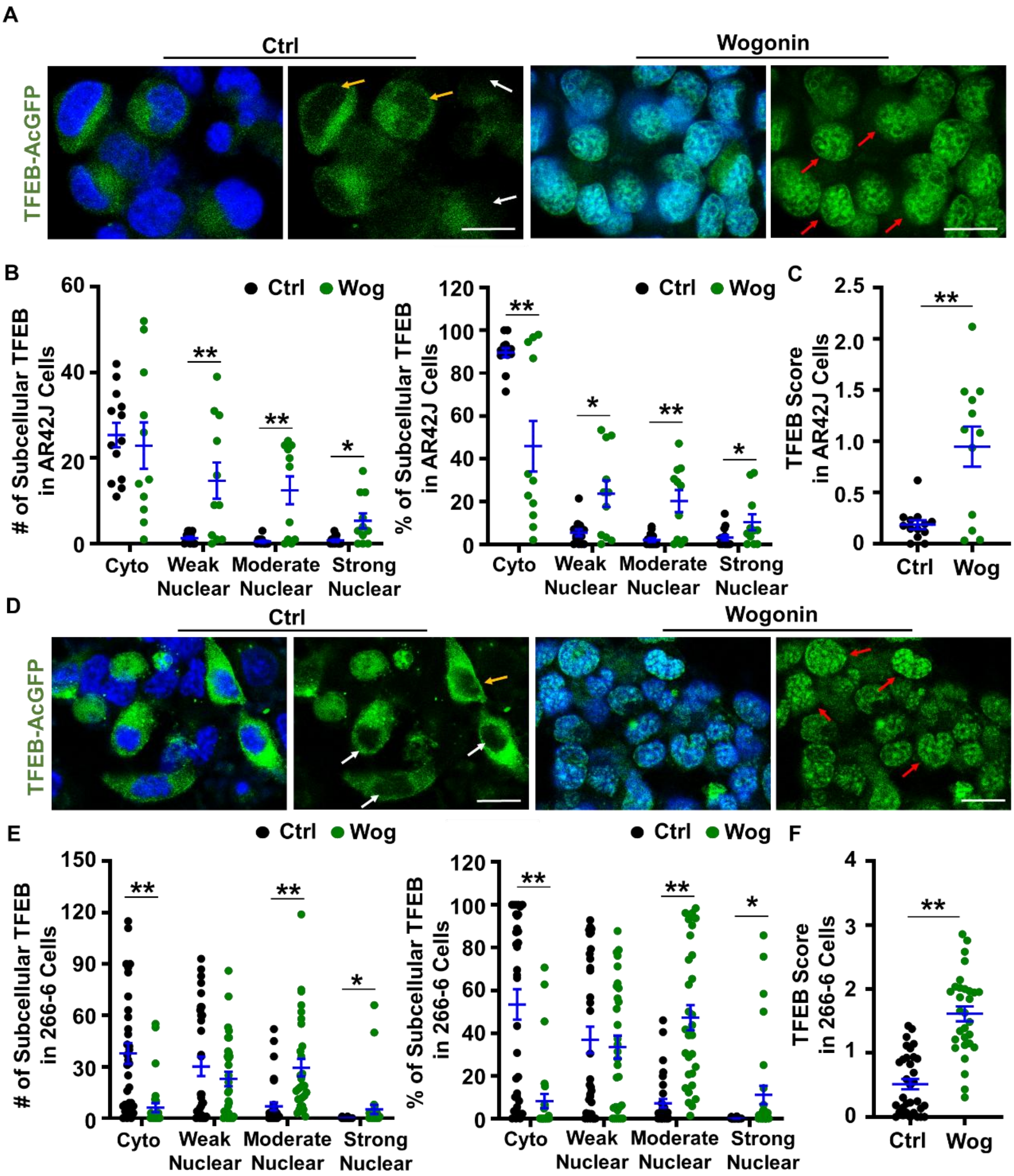

Supplementary Figure 9. Wogonin induces exogenous TFEB nuclear translocation in pancreatic cell lines. AR42J or 266-6 pancreatic cells were infected with TFEB-AcGFP lentivirus for 72h before treated with Wogonin. **(A)** AR42J cells were treated with 50 μM Wogonin for 6h then examined by confocal microscope. White, yellow or red arrow denotes weak, moderate or strong nuclear TFEB, respectively. Scale bar = 50 μm. **(B)** The number and percentage of cytosolic and nuclear TFEB were quantified, data shown are mean ± SEM (n

= 3). More than 360 cells were counted in each group. **p < 0.01, *p < 0.05 by Student t test. **(C)** TFEB score was calculated based on the subcellular distribution of TFEB, data shown are mean ± SEM (n = 3). **p < 0.01 by Student t test. **(D)** 266-6 cells were treated with 50 μM Wogonin for 6h then examined by confocal microscope. White, yellow or red arrow denotes weak, moderate or strong nuclear TFEB, respectively. Scale bar = 50 μm. **(E)** The number and percentage of cytosolic and nuclear TFEB were quantified, data shown are mean ± SEM (n = 3). More than 1900 cells were counted in each group. **p < 0.01, *p < 0.05 by Student t test. **(F)** TFEB score was calculated based on the subcellular distribution of TFEB, data shown are mean ± SEM (n = 3). **p < 0.01, *p < 0.05 by Student t test. **p < 0.01 by Student t test.

Supplementary references

1. Liu, Y., et al., *Wogonin effects on the efflux transporters BCRP and MRP2, explain its effectiveness in ulcerative colitis: Implications for metabolic and transport interactions*. Pharmacol Res, 2025. **212**: p. 107570.
