# Supplementary material for: Wogonin, a bioactive flavonoid from Scutellaria baicalensis, alleviates alcoholic pancreatitis via AMPK-mediated TFEB activation
Source: J Adv Res. 2025 Nov 10;86:891–907. doi: 10.1016/j.jare.2025.11.006 (PMC13453865; doi:10.1016/j.jare.2025.11.006)
Supplement: Supplementary file 2 [file mmc2.pdf]

Supplementary Table 1.

Binding kinetics of Wogonin to AMPK.

Ligand	Analyte	K_D (μM)	k_a (1/Ms)	k_d (1/s)	$χ^2$
AMPK	Wogonin	0.55 ± 0.02	$7.40*10^{5}$ ± $4.56*10^{4}$	$4.07*10^{-1}$ ± $1.84*10^{-2}$	<1
RSD (%)		5.08	10.67	7.81	

Data are mean ± SEM (n = 3).
