# Supplementary material for: Wogonin, a bioactive flavonoid from Scutellaria baicalensis, alleviates alcoholic pancreatitis via AMPK-mediated TFEB activation
Source: J Adv Res. 2025 Nov 10;86:891–907. doi: 10.1016/j.jare.2025.11.006 (PMC13453865; doi:10.1016/j.jare.2025.11.006)

Uncropped Fig. 1E

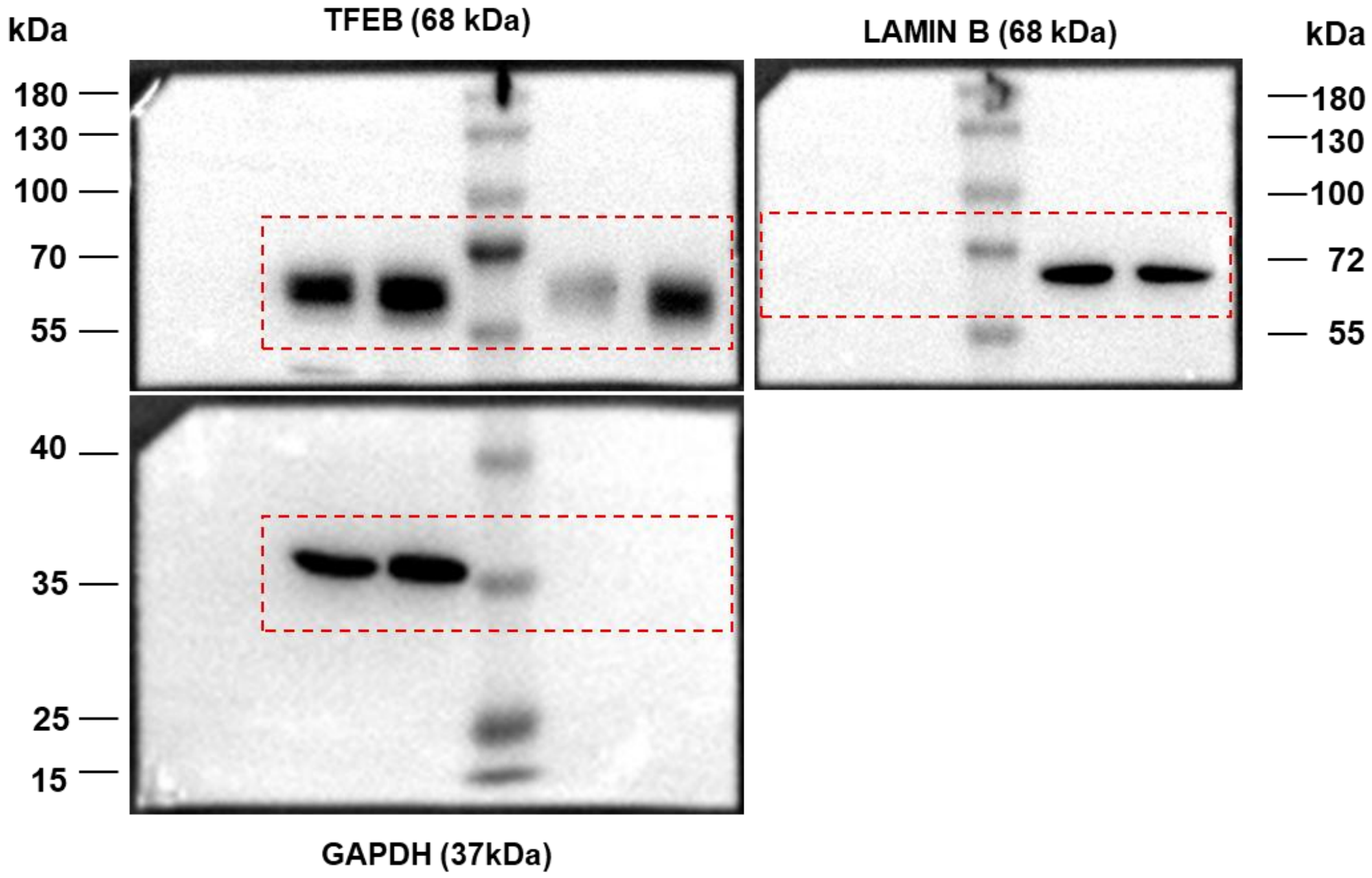

Uncropped Fig. 1G

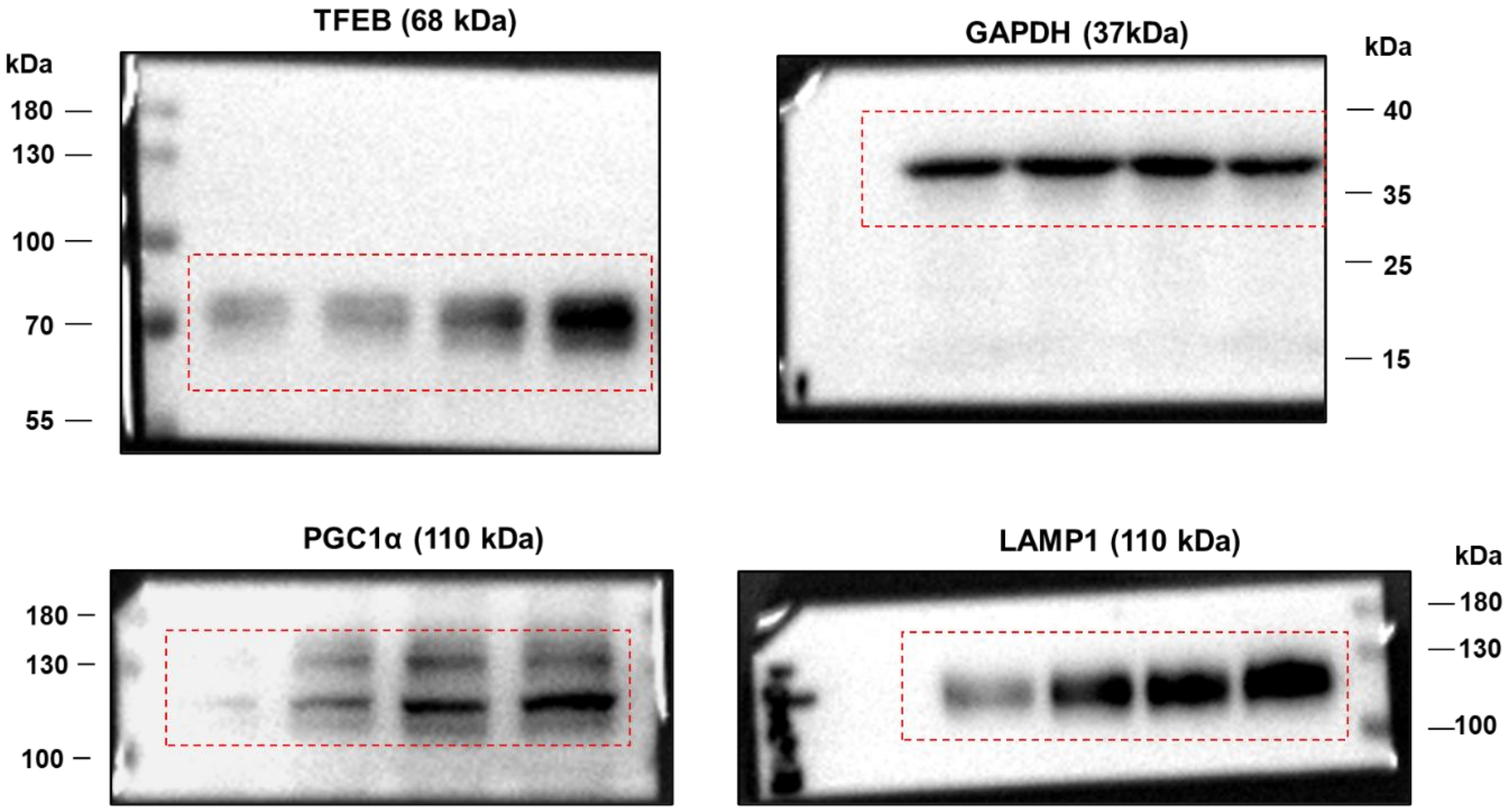

Uncropped Fig. 2C

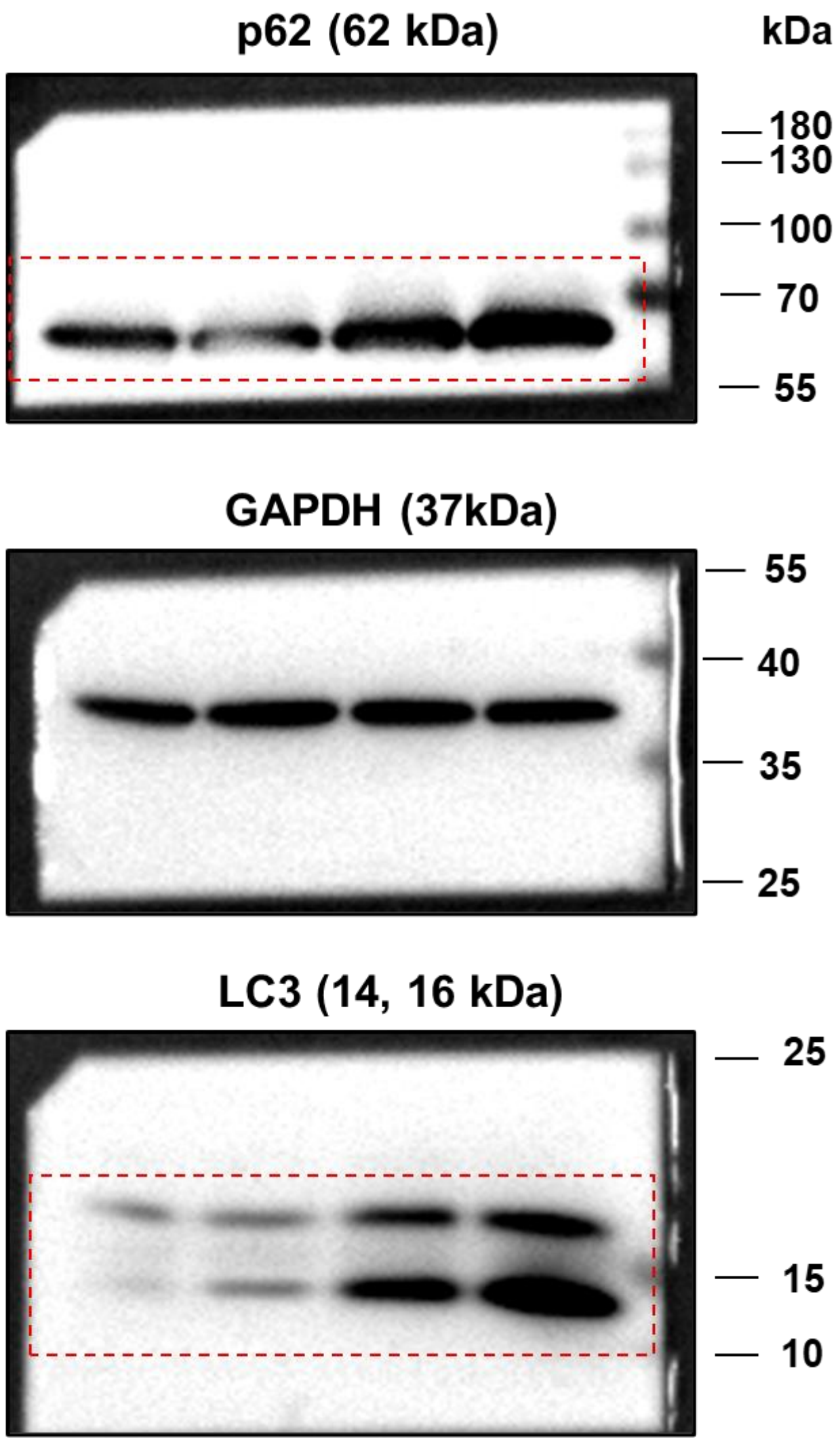

Uncropped Fig. 4A

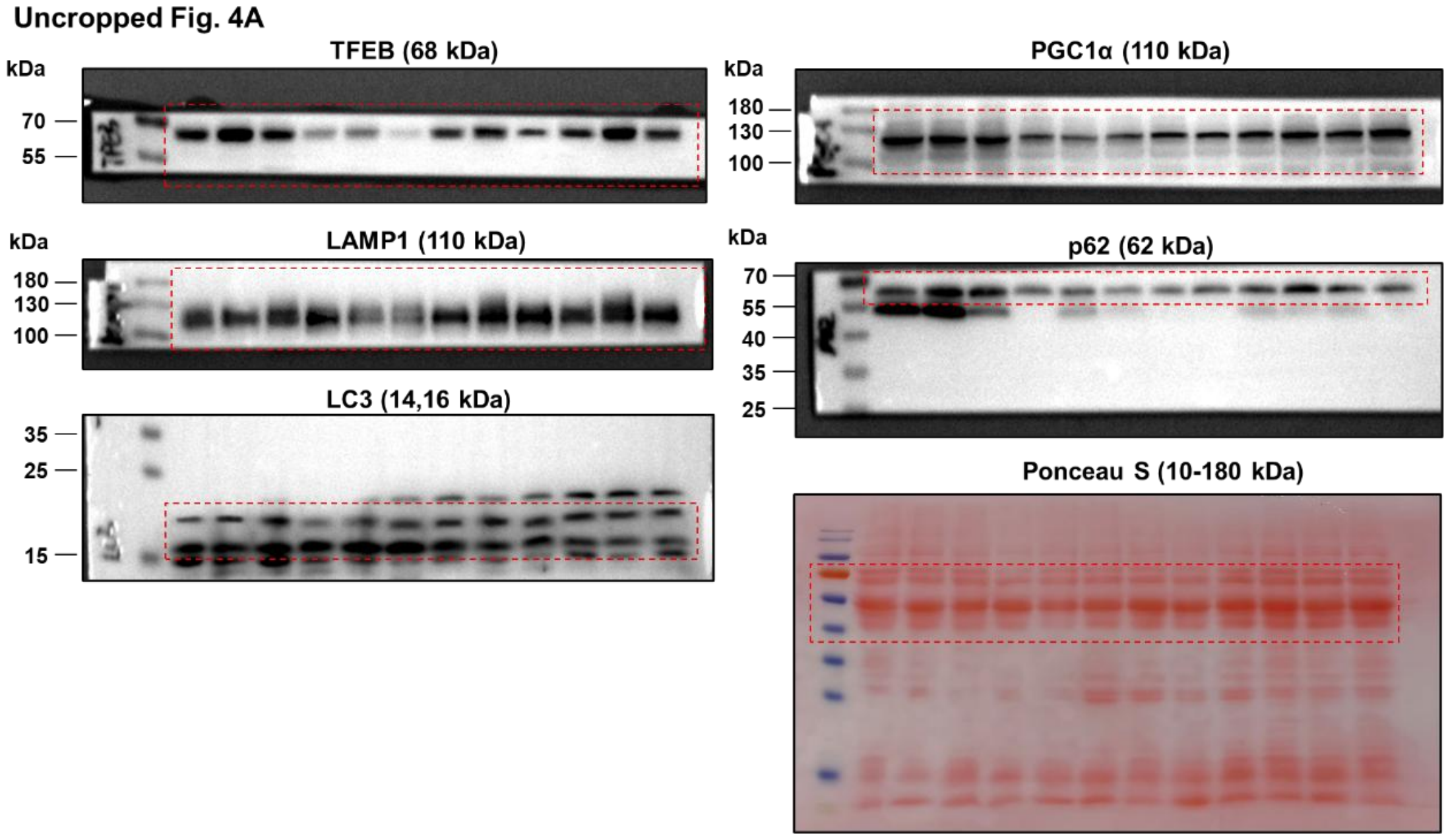

Uncropped Fig. 6B

kDa

TFEB (68 kDa)

Ponceau S (10-180 kDa)

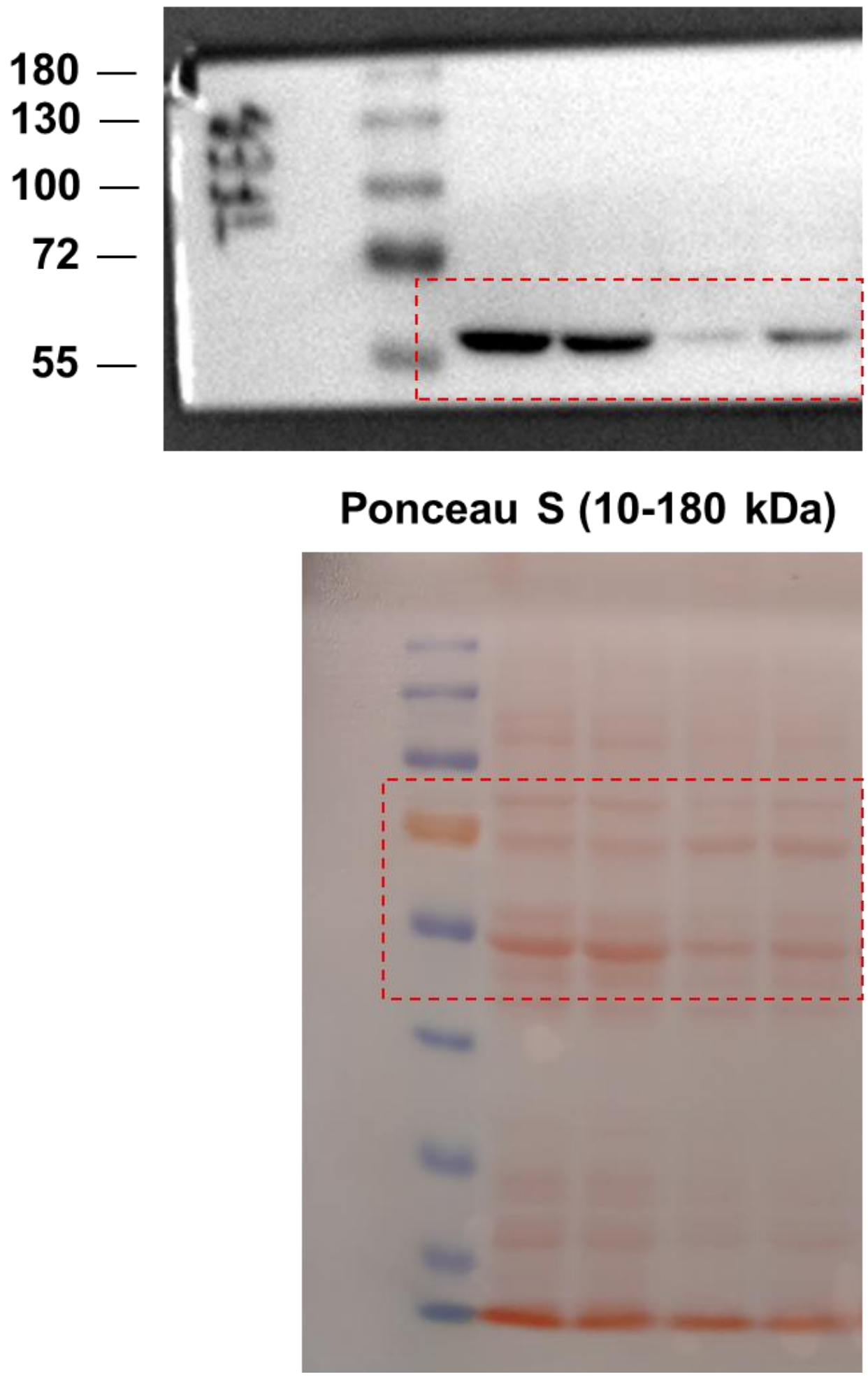

Uncropped Fig. 7D

p-AMPK^{T172} (62 kDa)

t-AMPK^{T172} (62 kDa)

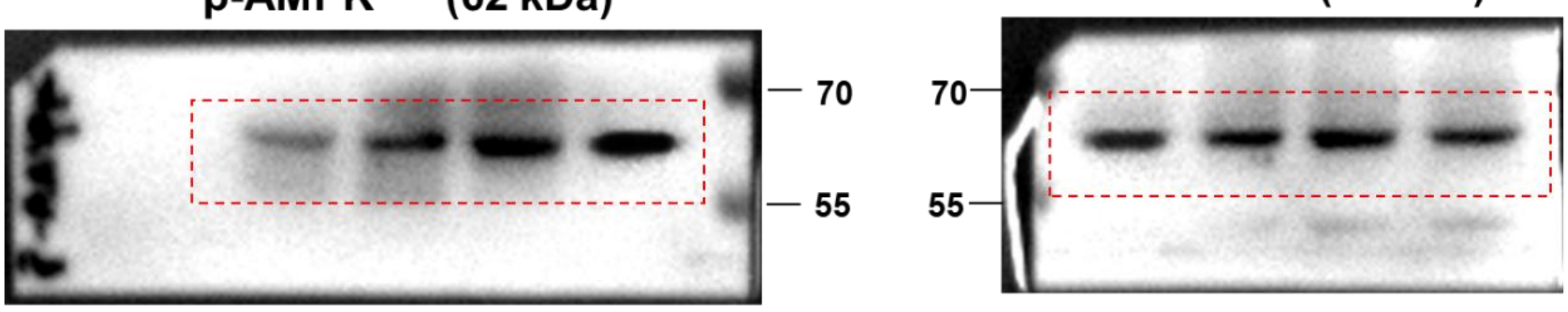

Uncropped Fig. 7F

p-AMPK^{T172} (62 kDa)

t-AMPK^{T172} (62 kDa)

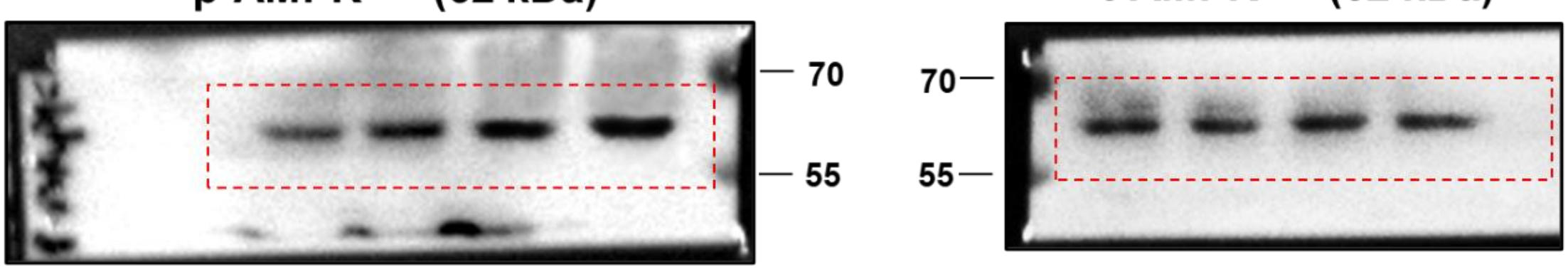

Uncropped Fig. 8E

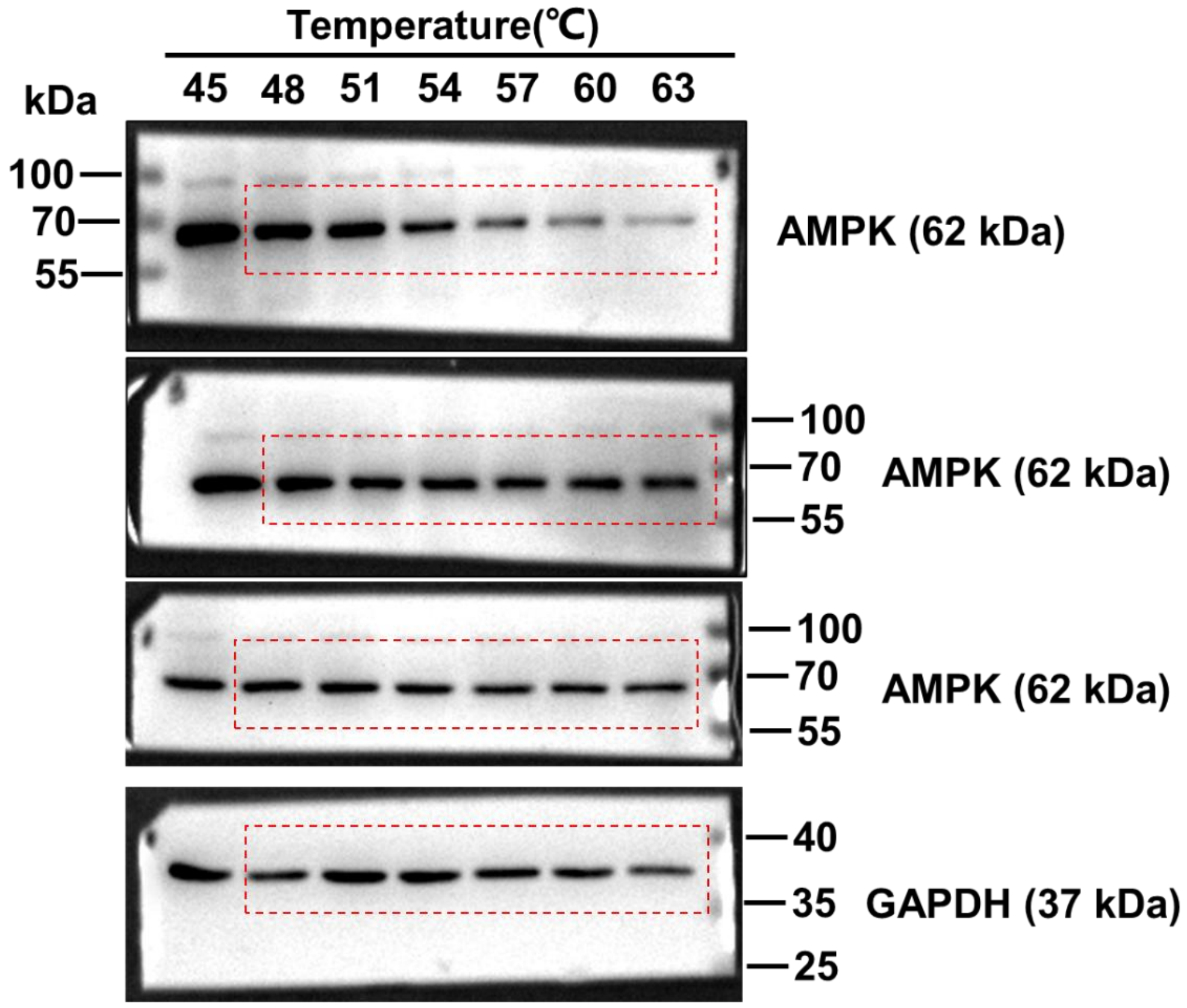

Uncropped Fig. 8G

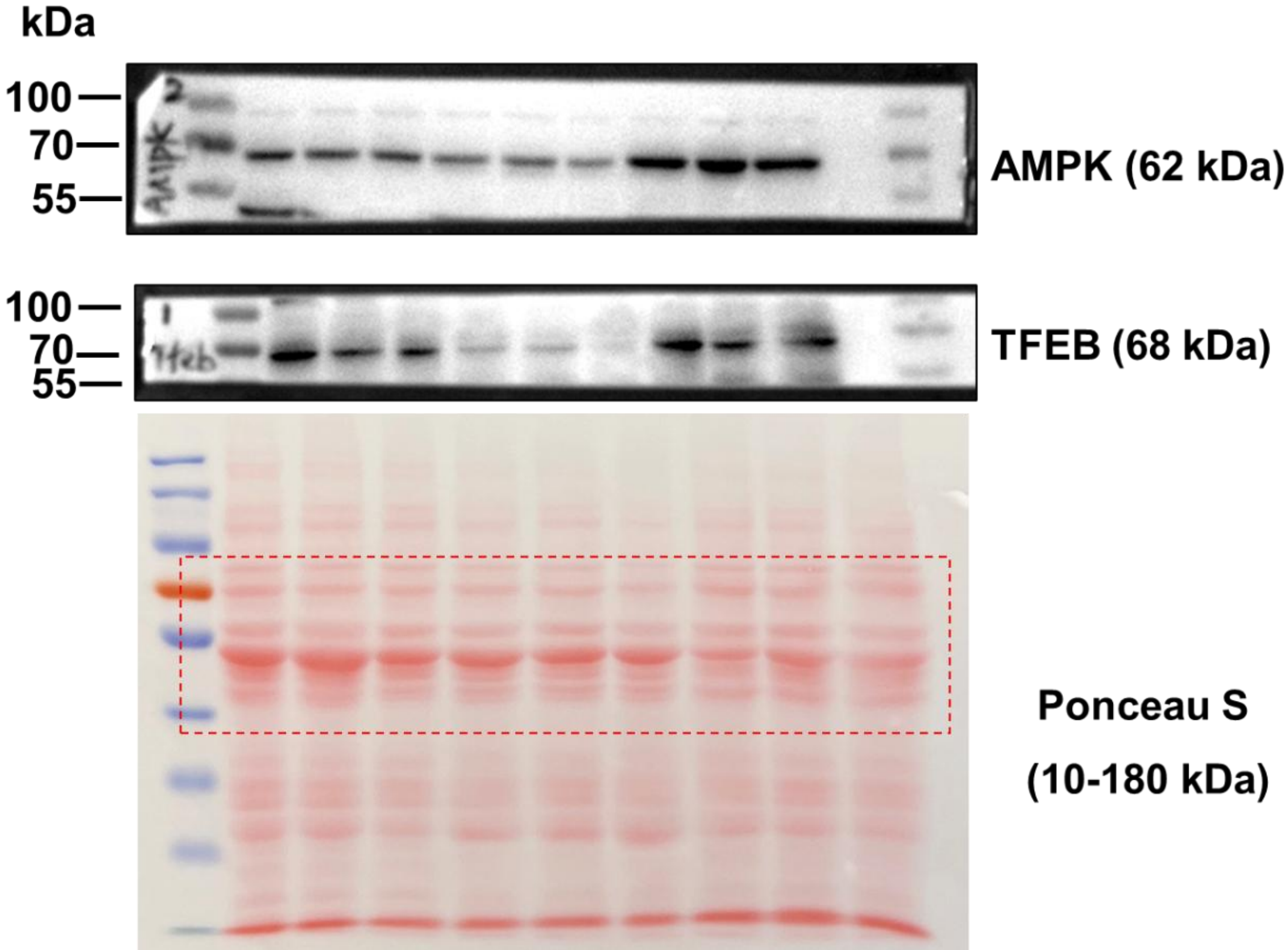

Uncropped Fig. 9E

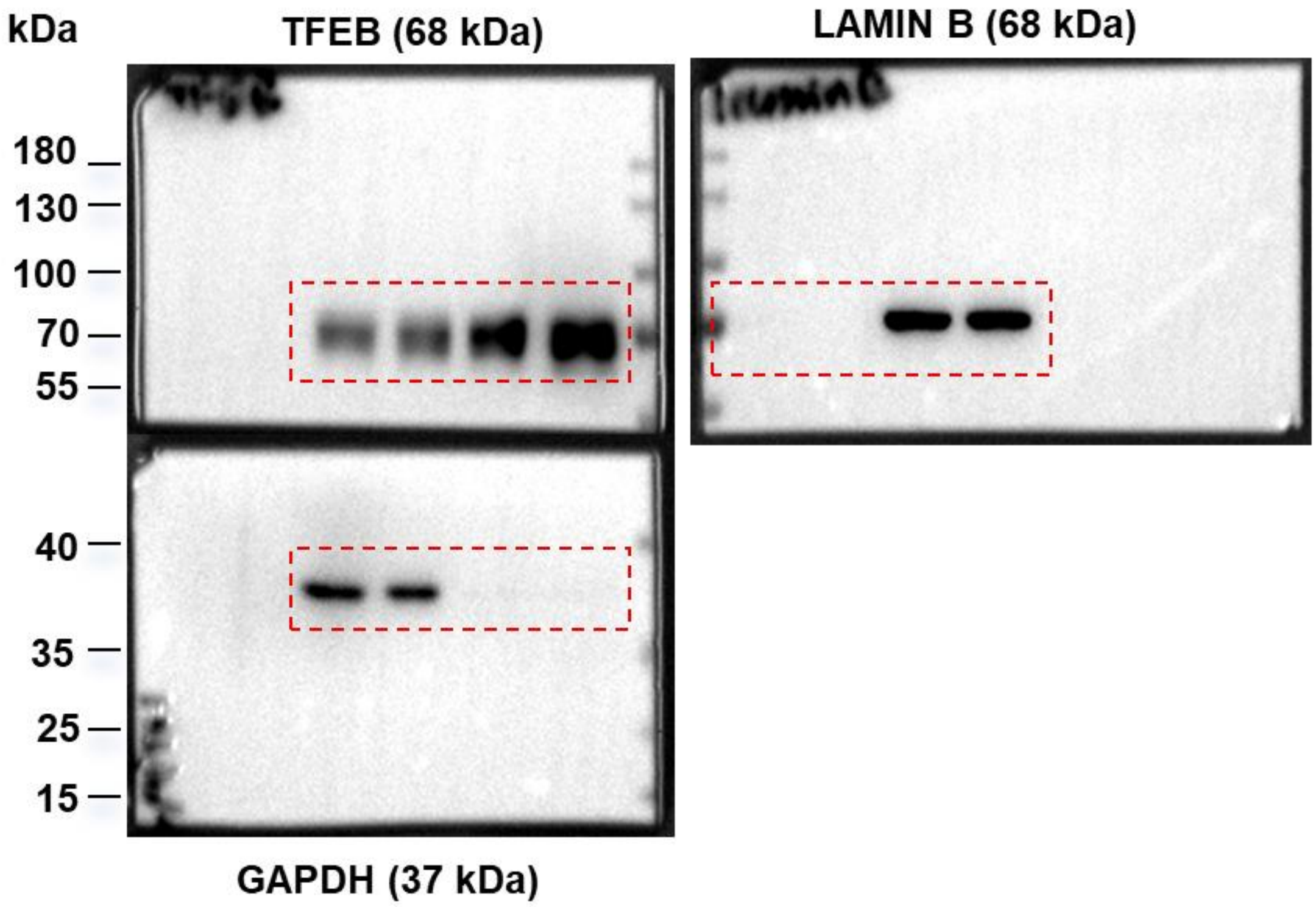

Uncropped Fig. 9G

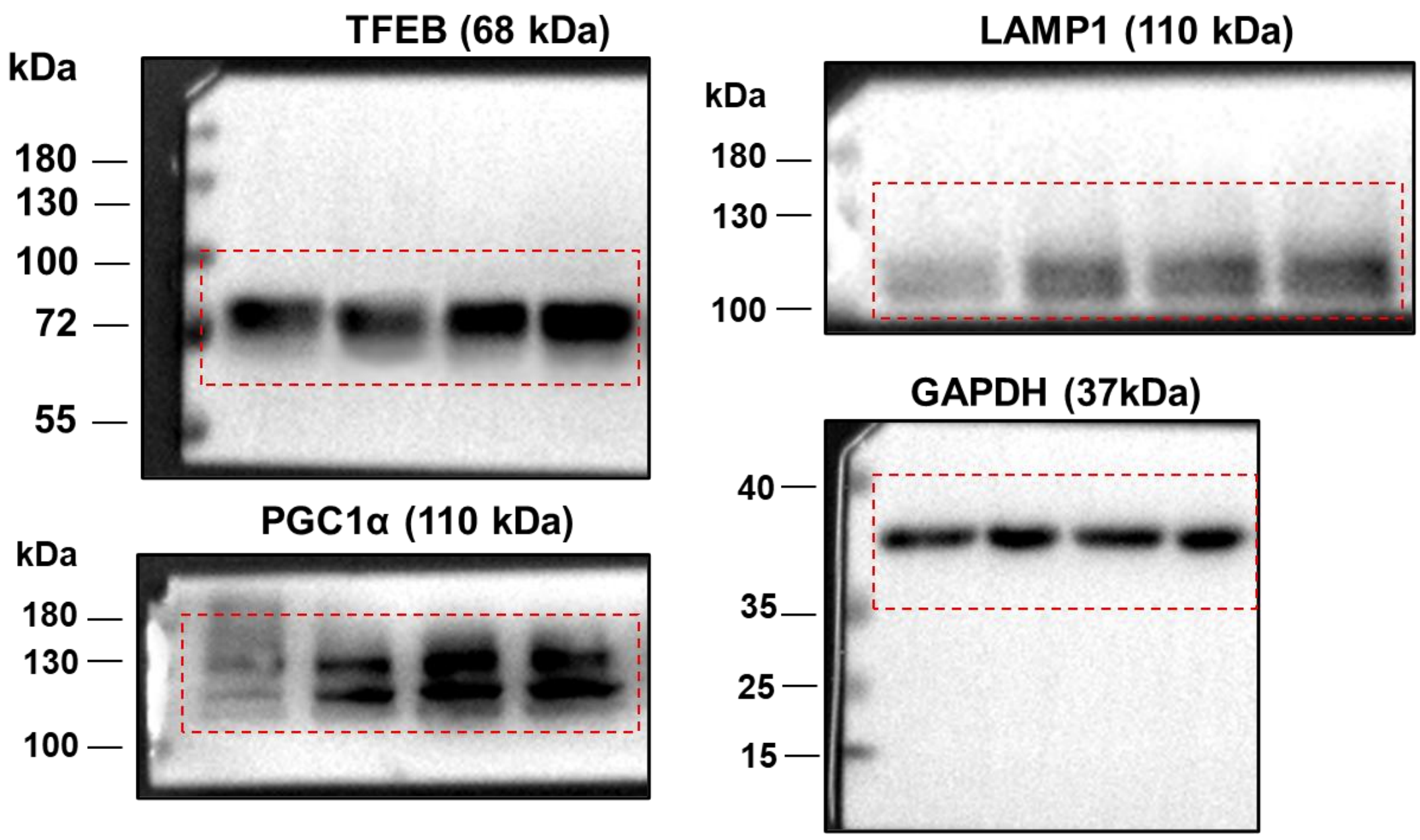

Uncropped Fig. 10C

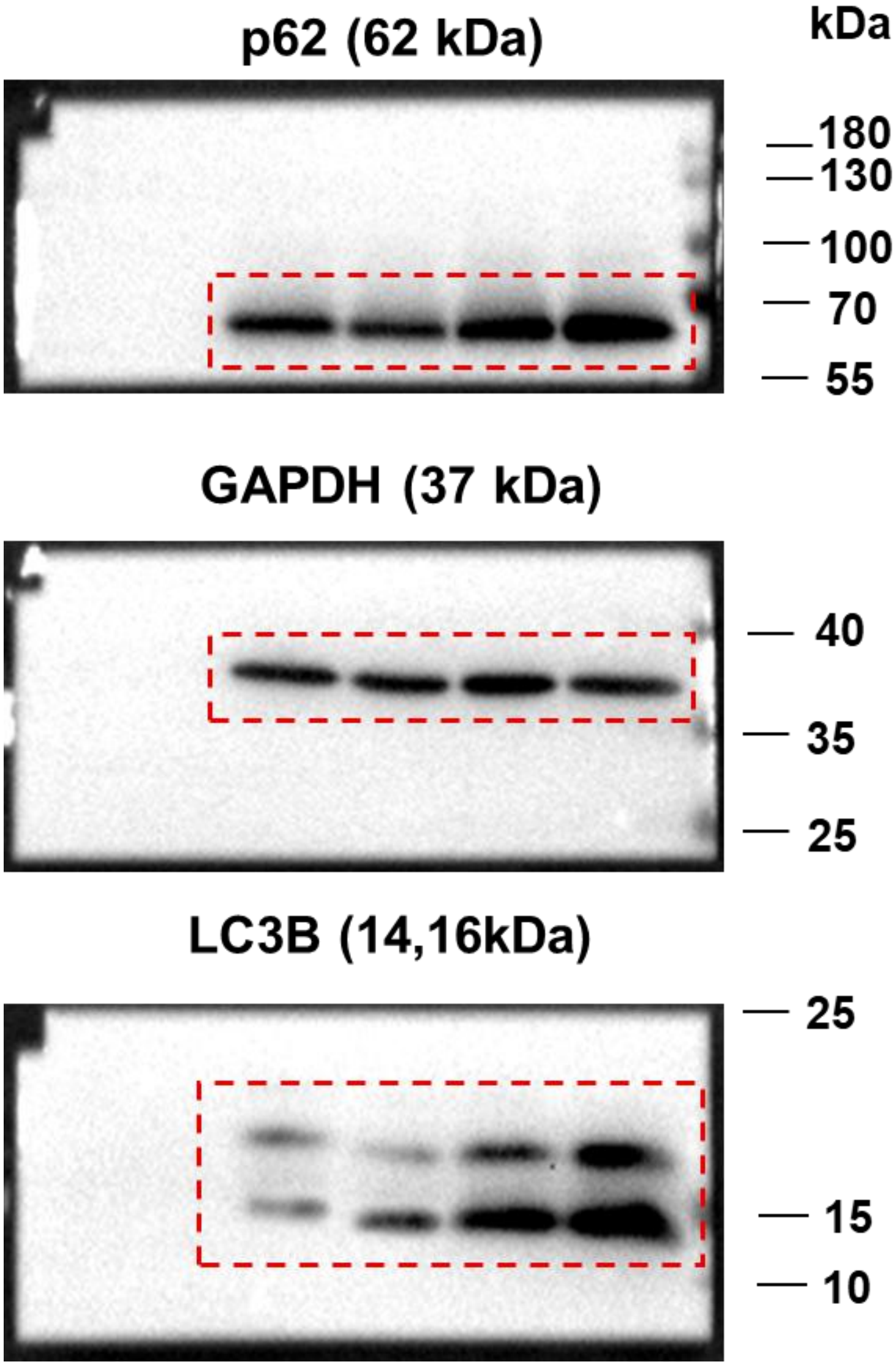

Supplement: Supplementary file 3 [file mmc4.pdf]
